# Dengue Virus NS1 Disrupts the Endothelial Glycocalyx, Leading to Hyperpermeability

**DOI:** 10.1371/journal.ppat.1005738

**Published:** 2016-07-14

**Authors:** Henry Puerta-Guardo, Dustin R. Glasner, Eva Harris

**Affiliations:** Division of Infectious Diseases and Vaccinology, School of Public Health, University of California, Berkeley, Berkeley, California, United States of America; Purdue University, UNITED STATES

## Abstract

Dengue is the most prevalent arboviral disease in humans and a major public health problem worldwide. Systemic plasma leakage, leading to hypovolemic shock and potentially fatal complications, is a critical determinant of dengue severity. Recently, we and others described a novel pathogenic effect of secreted dengue virus (DENV) non-structural protein 1 (NS1) in triggering hyperpermeability of human endothelial cells *in vitro* and systemic vascular leakage *in vivo*. NS1 was shown to activate toll-like receptor 4 signaling in primary human myeloid cells, leading to secretion of pro-inflammatory cytokines and vascular leakage. However, distinct endothelial cell-intrinsic mechanisms of NS1-induced hyperpermeability remained to be defined. The endothelial glycocalyx layer (EGL) is a network of membrane-bound proteoglycans and glycoproteins lining the vascular endothelium that plays a key role in regulating endothelial barrier function. Here, we demonstrate that DENV NS1 disrupts the EGL on human pulmonary microvascular endothelial cells, inducing degradation of sialic acid and shedding of heparan sulfate proteoglycans. This effect is mediated by NS1-induced expression of sialidases and heparanase, respectively. NS1 also activates cathepsin L, a lysosomal cysteine proteinase, in endothelial cells, which activates heparanase via enzymatic cleavage. Specific inhibitors of sialidases, heparanase, and cathepsin L prevent DENV NS1-induced EGL disruption and endothelial hyperpermeability. All of these effects are specific to NS1 from DENV1-4 and are not induced by NS1 from West Nile virus, a related flavivirus. Together, our data suggest an important role for EGL disruption in DENV NS1-mediated endothelial dysfunction during severe dengue disease.

## Author Summary

Dengue is the most prevalent mosquito-borne disease in humans and represents a major public health problem worldwide. Leakage of fluids and molecules from the bloodstream into tissues can lead to shock and potentially death and is a critical determinant of dengue disease severity. Recently, we showed that a secreted protein from dengue virus (DENV)-infected cells, non-structural protein 1 (NS1), can trigger increased leakage both in human cell culture and mouse models. It has been shown that NS1 can activate toll-like receptor 4 on peripheral blood mononuclear cells, leading to secretion of pro-inflammatory cytokines that can result in vascular leak. However, the mechanism by which NS1 triggers hyperpermeability directly in human endothelial cells remained undefined. The endothelial glycocalyx layer (EGL) is a network of membrane-bound molecules that lines endothelial cells on the inside of blood vessels, helping to regulate proper vascular function. Here, we show that DENV NS1 can disrupt the integrity of the EGL, inducing breakdown and shedding of key components. This is mediated by NS1 induction of cellular enzymes (e.g., sialidases, heparanase, and cathepsin L) that contribute to EGL alterations. Inhibitors that block these enzymes prevent both EGL disruption and endothelial permeability. These effects were all demonstrated to be specific to NS1 from DENV serotypes 1–4, as NS1 from the related West Nile Virus did not produce EGL alterations or increased leakage. Our study suggests a novel role for DENV NS1 in inducing EGL disruption to increase fluid leakage during severe dengue disease.

## Introduction

The four dengue virus serotypes (DENV1-4) are mosquito-borne flaviviruses that are responsible for ~390 million infections per year worldwide [1]. Of these, up to 96 million manifest in clinical disease. The majority of these cases are dengue fever (DF), the uncomplicated form of disease. However, a subset develop severe dengue disease, including dengue hemorrhagic fever (DHF) and dengue shock syndrome (DSS), characterized by increased vascular leak, leading to shock and potentially death [2]. Pleural effusion resulting in respiratory distress is one of the most common signs of plasma leakage in DHF/DSS cases [3].

Vascular hyperpermeability arises as a result of endothelial barrier dysfunction, leading to increased passage of fluids and macromolecules across the endothelium. Traditionally, tight and adherens junctions have been considered to be the primary determinants of endothelial barrier function [4]. Over the past few years, however, the endothelial glycocalyx layer (EGL) has been recognized as a key regulator of vascular permeability [5]. The EGL is a network of glycoproteins bearing acidic oligosaccharides and terminal sialic acid (*N*-acetyl-neuraminic acid, Sia), as well as membrane-bound proteoglycans associated with glycosaminoglycan (GAG) side chains including heparan sulfate (HS), hyaluronic acid, and chondroitin sulfate [6]. The EGL extends along the endothelial layer coating the luminal surface of blood vessels.

Secondary DENV infection with a serotype distinct from the first DENV infection is a known risk factor for severe dengue disease. Several hypotheses have been proposed to explain severe dengue disease, including poorly neutralizing, cross-reactive antibodies and exacerbated T cell responses that together lead to production of vasoactive cytokines, causing vascular leakage that can result in shock [7]. Another potential component is DENV nonstructural protein 1 (NS1), a glycosylated 48 kDa protein that is the only viral protein secreted from infected cells, with high concentrations circulating in the blood of patients with severe dengue disease. NS1 plays a role in viral replication, immune evasion, and pathogenesis via activation of complement pathways [8]. More recently, we and others demonstrated that DENV NS1 alone can trigger endothelial hyperpermeability, resulting in vascular leakage [9, 10]. Modhiran et al. [10] showed that NS1 acts as a pathogen-associated molecular pattern (PAMP), activating mouse macrophages and human peripheral blood mononuclear cells (PBMCs) via toll-like receptor 4 (TLR4) to secrete pro-inflammatory cytokines such as tumor necrosis factor–α (TNF-α), interleukin-6 (IL-6), interferon-β (IFN-β), IL-1β, and IL-12. This effect was inhibited by a TLR4 antagonist (LPS-RS) and an anti-TLR4 antibody [10]. Further, we found that inoculation of mice with NS1 alone causes increased vascular leakage and induction of pro-inflammatory cytokines (TNF-α, IL-6), while NS1 combined with a sub-lethal DENV inoculum results in a lethal vascular leak syndrome [9]. Our *in vitro* experiments showed that NS1 also increases the permeability of human endothelial cells [9]. The increased permeability *in vitro*, as well as mortality in mice, was prevented by administration of NS1-immune polyclonal mouse sera or anti-NS1 monoclonal antibodies [9]. Likewise, immunization with recombinant NS1 from each of the four DENV serotypes protected against lethal challenge in the vascular leak model [9].

However, the mechanism by which DENV NS1 stimulates endothelial cells to induce vascular leak is poorly understood. NS1 has been proposed to bind to heparan sulfate on the surface of endothelial cells [11], but how this interaction leads to an increase in endothelial permeability has not been described. Therefore, we evaluated whether NS1 triggers disruption of the EGL and defined the mechanism through which this occurs.

## Results

### Binding of DENV2 NS1 to endothelial cells induces endothelial hyperpermeability

**Fig 1:**
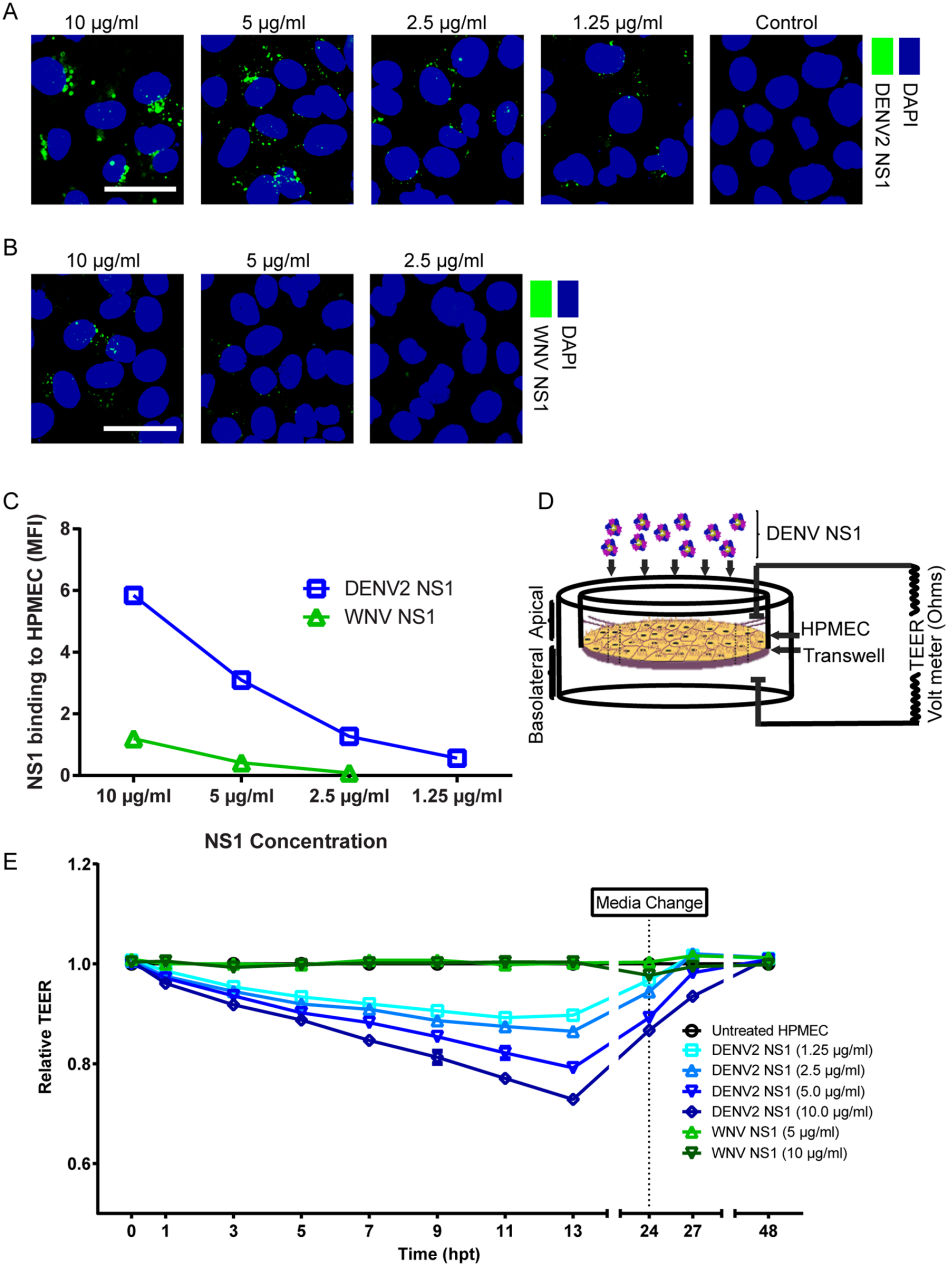
DENV2 but not WNV NS1 binding to endothelial cells induces endothelial barrier dysfunction. **(A-B)** Binding of DENV2 NS1 **(A)** and WNV NS1 **(B)** proteins, examined by confocal microscopy. NS1 is stained in green, and nuclei are stained with *Hoechst* (blue). Images (20X) are representative of three individual experiments. Scale bars, 10 μM. A trace of the mean fluorescence intensity (MFI) of one representative field is shown below each image. **(C)** The amount of NS1 bound to HPMEC monolayers in (A) and (B) is quantified and expressed as MFI. Graph shows average of quantification from three independent experiments. DENV2 NS1 binding to HPMEC monolayers is significantly higher than WNV NS1 binding at all concentrations (p<0.0001). **(D)** Experimental schematic of trans-endothelial electrical resistance (TEER) experiments. **(E)** TEER assay to evaluate the effect of DENV2 and WNV NS1 proteins on HPMEC endothelial permeability at indicated concentrations. Relative TEER values from three independent experiments performed in duplicate are plotted at the indicated time points. Error bars indicate standard error of the mean (SEM). All DENV2 NS1 concentrations induce statistically significant decreases in TEER (p<0.0001).

Soluble DENV2 NS1 attaches to the surface of human endothelial cells, especially pulmonary microvascular endothelial cells [11]. In severe dengue disease, major accumulation of fluids occurs in the pleura (pleural effusion), a thin membrane that lines the surface of the lungs [12]. This suggests that the lung represents an important site of endothelial barrier dysfunction characteristic of severe dengue. In this study, we used an *in vitro* model of endothelial permeability to initially examine the ability of soluble NS1 from DENV serotype 2 and West Nile virus (WNV NS1) to interact with cultured human pulmonary microvascular endothelial cells (HPMEC). In the first experiment, we found that DENV2 NS1 showed dose-dependent binding (1.25–10 μg/ml) to HPMEC monolayers at one hour post-treatment (hpt) (Fig 1A and 1C). In contrast, WNV NS1 (2.5–10 μg/ml), from a closely-related member of the *Flavivirus* genus, displayed significantly less binding (Fig 1B and 1C). A time course for DENV2 NS1 (5 μg/ml) attachment to the surface of HPMEC showed a maximum peak for NS1 staining between 3 and 12 hpt; no NS1 could be detected on the surface of HPMEC after 24 hpt (S1A and S1B Fig). This NS1 binding pattern reflected decreased trans-endothelial electrical resistance (TEER) observed in HPMEC and other endothelial cell lines, including primary human umbilical vein endothelial cell (HUVEC) [9] and human dermal microvascular endothelial cell (HMEC-1) monolayers exposed to DENV2 NS1 (Figs 1D and 1E and S2A). Increased endothelial permeability is induced by NS1 from DENV1-4 [9] after 3 hpt in a dose-dependent manner, and the effect persists for more than 12 hours (Fig 1E). Although we previously reported [9] that all NS1 proteins tested negative for bacterial endotoxin using the Endpoint Chromogenic Limulus Amebocyte Lysate (LAL) QCL-1000TM kit (Lonza) (<0.1 EU/ml per 25 mg of protein), we included an additional test using DENV2 NS1 pre-treated with the LPS-binding antibiotic polymyxin B (25 μg/ml). Polymyxin B did not inhibit DENV2 NS1-induced endothelial hyperpermeability in HPMEC, further supporting that this effect is specific to DENV2 NS1 and is not due to residual LPS contamination (S2B Fig).

### DENV2 NS1 induces degradation of sialic acid in the EGL of endothelial cells

**Fig 2:**
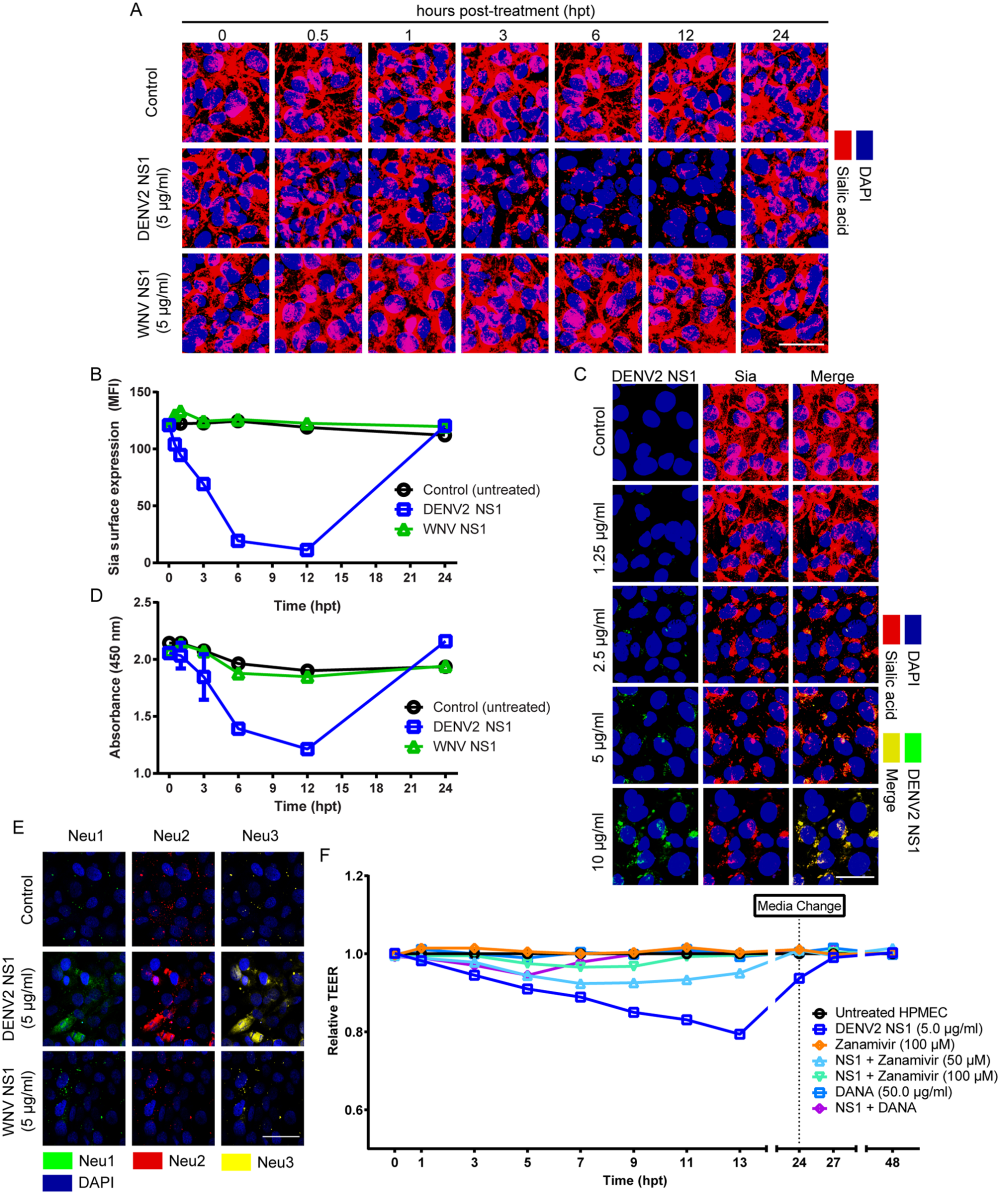
DENV2 but not WNV NS1 modulates the expression of Sia in the EGL of HPMEC. **(A)** Sia expression on HPMEC monolayers after treatment with DENV2 or WNV NS1 (5 μg/ml), examined by confocal microscopy. Sia was stained with WGA-A647 (red) at indicated time points (hpt). Nuclei stained with *Hoechst* (blue). Images (20X) and MFI values are representative of three independent experiments. Scale bar, 10 μM. **(B)** Quantification of MFI in (A) from three independent experiments. Sia expression in DENV2 NS1-treated monolayers is significantly different from WNV-treated and untreated controls from 0.5 to 12 hpt (p<0.0001). **(C)** DENV2 NS1 (green) induces dose-dependent reduction of Sia staining (red) on HPMEC after 3 hpt. Untreated cells were used as a positive control for Sia expression. **See also** S1 Fig. **(D)** ELISA to detect free Sia released into culture supernatant of HPMEC over time (hpt) under indicated experimental conditions. DENV2 NS1 shedding of Sia is significantly lower than WNV-treated and untreated controls at 6 and 12 hpt (p<0.0005). **(E)** Endothelial sialidase (Neu1, Neu2, Neu3) expression in HPMEC monolayers after treatment with DENV2 or WNV NS1 (5 μg/ml) at 3 hpt. Neu1, Neu2, and Neu3 are stained in green, red, and yellow, respectively. **(F**) Effect of Zanamivir and 2,3-didehydro-2-deoxy-N-acetylneuraminic acid (DANA) on DENV2 NS1-mediated endothelial hyperpermeability (TEER) in HPMEC monolayers. Relative TEER values from three independent experiments performed in duplicate are plotted at indicated time points (hpt). TEER values for monolayers treated with DENV2 NS1 combined with Zanamivir (50, 100 μM) or DANA (50 μg/ml) are significantly different from monolayers treated with DENV2 NS1 alone (p<0.0001). Error bars indicate SEM throughout.

The EGL on the surface of the endothelium plays an important role in several cellular functions, including cell-to-cell communication, cell-matrix interaction, and vascular homeostasis [5], and a mature EGL has been shown to exist on cultured HPMEC *in vitro* [13]. To examine the effect of flavivirus NS1 proteins on the integrity of the EGL, HPMEC monolayers were exposed to DENV2 or WNV NS1 (5 μg/ml), in the range of NS1 concentrations seen in severe dengue in humans [14, 15]. The expression of Sia, a major component of the EGL [16], was visualized using the lectin wheat germ agglutinin (WGA) conjugated to Alexa 647 [17, 18]. WGA has been described to not only bind to Sia but also to *N*-acetylglucosamine (Molin et al., 1986), another monosaccharide expressed on the surface of endothelial cells. In this study, abundant binding of WGA at 30 minutes (min) and 1 hpt reflects normal distribution of Sia residues on the surface of HPMEC (Fig 2A). In contrast, HPMEC monolayers treated with exogenous neuraminidase (0.5 UI, *Clostridium perfringens*, Sigma), which specifically cleaves Sia, almost completely eliminated WGA staining, indicating that WGA binds most abundantly to Sia on the surface of HPMEC (S3A and S3B Fig). The homogenous distribution of Sia observed in untreated HPMEC was significantly disrupted in a dose-dependent manner 3–12 h after addition of DENV2 NS1 but not WNV NS1 (Fig 2A, 2B and 2C). This same effect was observed in both HUVEC and HMEC-1 exposed to DENV2 NS1 after 3 and 6 h (S3C and S3D Fig). Normal distribution of Sia was re-established by 24 hpt (Fig 2A and 2B). Binding of DENV2 NS1 to HPMEC appeared to co-localize with the WGA staining of Sia residues in the EGL, suggesting that DENV2 NS1 may use Sia-linked glycans as adhesion molecules to mediate NS1-endothelial cell surface interaction (Figs 2C and S1C). Next, to examine whether Sia was degraded or released from the surface of HPMEC exposed to DENV2 NS1, we assessed the presence of free Sia in cultured HPMEC supernatant using a specific Sia immunoassay. Supernatant collected from endothelial monolayers treated with DENV2 NS1 showed a significant time-dependent decrease in Sia levels compared with supernatant from untreated cells and WNV NS1-treated monolayers (Fig 2D), indicating Sia is not being released into the medium of DENV2 NS1-treated HPMEC. Interestingly, expression of Neu1, Neu2, and Neu3, three mammalian sialidases found in endothelial cells, was strongly increased in HPMEC monolayers treated with DENV2 NS1 but not WNV NS1 at 3 hpt, potentially contributing to Sia degradation (Fig 2E). To determine the functional significance of DENV2 NS1-triggered disruption of Sia in the EGL, sialidase activity was inhibited using Zanamivir, an influenza neuraminidase inhibitor that has been shown to significantly inhibit Neu2 and Neu3 [19], and 2-deoxy-2,3-didehydro-N-acetyl-neuraminic acid (DANA), a transition state analog inhibitor of influenza virus neuraminidase found to be active against mammalian Neu3 [20]. Both Zanamivir (50, 100 μM) and DANA (25 μg/ml) partially protected HPMEC monolayers from DENV2 NS1-mediated endothelial hyperpermeability as measured by TEER (Fig 2F), indicating that alteration of Sia distribution on the surface of HPMEC induced by DENV2 NS1 contributes to increased permeability.

### DENV2 NS1 induces shedding of heparan sulfate proteoglycans from the EGL

**Fig 3:**
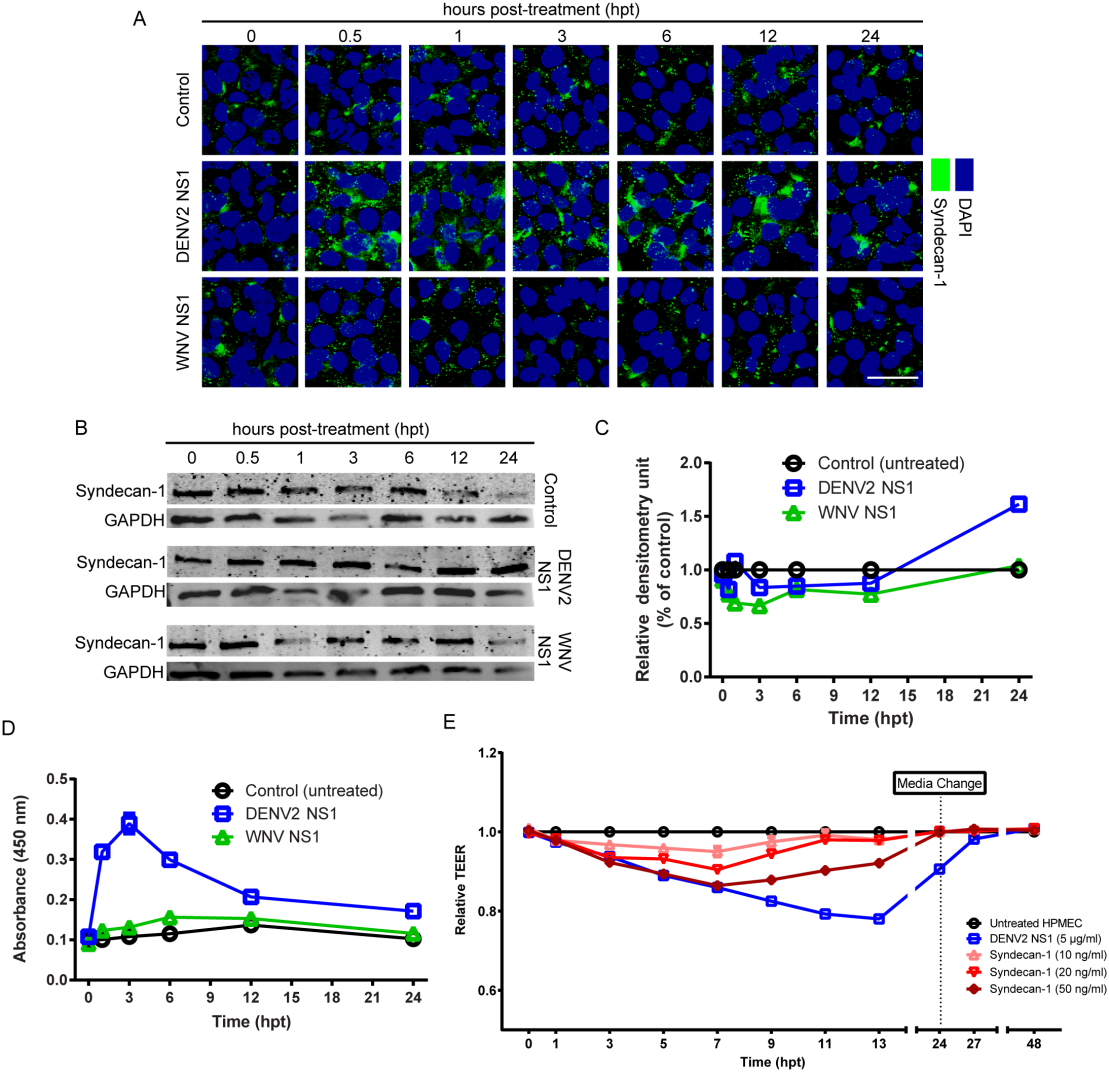
DENV2 NS1 increases the surface staining of syndecan-1 in the EGL of HPMEC. **(A)** Staining of syndecan-1 (green) on the surface of HPMEC monolayers over time (hpt) after treatment with DENV2 or WNV NS1 proteins (5 μg/ml), examined by confocal microscopy. Untreated cells were used as a control for basal syndecan-1 detection. Nuclei are stained with *Hoechst* (blue). Images are representative of three individual experiments (20X). Scale bar, 10 μM. A trace of the MFI for DENV2 NS1 of one representative field is shown below each image. **See also** S4A Fig. **(B)** Expression of syndecan-1 in total protein extracts at indicated time points (hpt) in the presence of DENV2 or WNV NS1 proteins (5 μg/ml), as measured by Western blot. Ten μg/ml of total protein was loaded, and GAPDH expression was used as protein loading control. **(C)** Densitometry data normalized to control untreated cells. **(D)** Levels of syndecan-1 shed from the surface of HPMEC after treatment with DENV2 or WNV NS1 proteins (5 μg/ml) as measured by ELISA from three independent experiments. Surface staining of syndecan-1 is significantly higher in monolayers treated with DENV2 NS1 than in WNV-treated and untreated controls (p<0.0005). **(E)** Effect of recombinant syndecan-1 on TEER of HPMEC monolayers. Relative TEER values from three independent experiments performed in duplicate are plotted at indicated time points. All concentrations of syndecan-1 induce significant decreases in TEER value (10 ng/ml: p <0.05; 20 ng/ml, 50 ng/ml: p<0.0001). Error bars indicate SEM throughout.

In addition to Sia, the EGL contains a large variety of heparan sulfate proteoglycans (HSPGs) [21, 22], including syndecans, which consist of a core protein modified by HS chains [23]. Syndecan-1 is considered the primary syndecan of endothelial cells, including the vascular endothelium [24]; thus, alteration of its expression or distribution can affect the integrity of the EGL as well as endothelial barrier function [25]. The expression and distribution of syndecan-1 was evaluated on HPMEC stimulated with DENV2 NS1 or WNV NS1. WNV NS1 did not modify the distribution of syndecan-1 on HPMEC monolayers, but treatment with DENV2 NS1 resulted in increased staining of syndecan-1 starting 30 min post-treatment and persisting for more than 12 h (Figs 3A and S4A). Similar results were observed for the extracellular matrix (ECM) HSPG perlecan (S4B Fig). However, syndecan-1 levels were similar in HPMEC treated with DENV2 NS1 or WNV NS1 compared to untreated controls, as detected by Western blot (Fig 3B and 3C). At 24 hpt, a small increase of syndecan-1 protein levels was detected by confocal microscopy and Western blot in DENV2 NS1-treated cells compared to control and WNV NS1-treated cells. Using an immunoassay for detection of soluble syndecan-1 ectodomain, increased levels of syndecan-1 ectodomain were found in conditioned media from DENV2 NS1-stimulated HPMEC at 1–24 hpt compared to untreated and WNV NS1-treated HPMEC (Fig 3D). Notably, recombinant syndecan-1 alone was able to increase the permeability of HPMEC monolayers in a dose-dependent manner (Fig 3E), suggesting that syndecan-1 shed from HPMEC after DENV2 NS1 stimulation may be involved in modulating endothelial barrier function.

### DENV2 NS1 increases activity of cathepsin L and its activation of heparanase in endothelial cells

**Fig 4:**
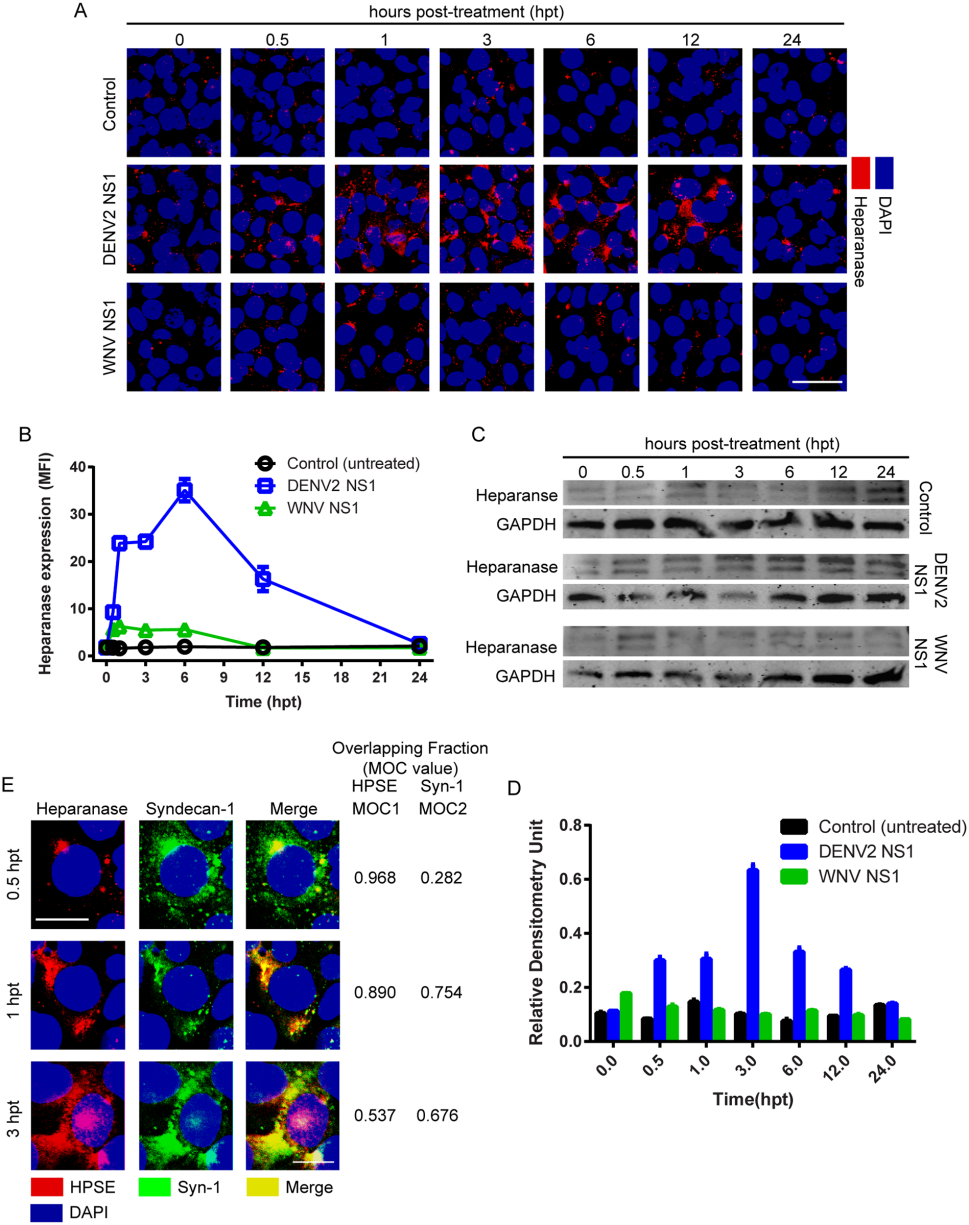
DENV2 NS1 triggers the activation/increased expression of endothelial heparanase. **(A)** Heparanase expression (red) in HPMEC monolayers over time (hpt) after treatment with DENV2 or WNV NS1 proteins (5 μg/ml), examined by confocal microscopy. Untreated cells were used as a control for basal heparanase expression. Nuclei stained with *Hoechst* (blue). Images are representative of three individual experiments (20X). Scale bar, 10 μM. A trace of the mean fluorescence intensity (MFI) for DENV2 NS1 of one representative field is shown below each image. **(B)** Quantification of MFI in (A) from three independent experiments. Staining of heparanase in monolayers treated with DENV2 NS1 is significantly different from WNV-treated and untreated controls from 0.5–12 hpt (p<0.0001). **(C)** Heparanase activation in HPMEC monolayers at indicated time points (hpt) after interaction with DENV2 and WNV NS1 proteins (5 μg/ml) via Western blot. Ten μg/ml of total protein was loaded, and GAPDH expression was used as a protein loading control. Upper (~60 kDa) and lower bands (~50 kDa) correspond to inactive and active forms of human heparanase I, respectively. **(D)** Densitometry of Western blot from (C). Graph shows lower band densitometries corresponding to active heparanase (~50 kDa), normalized to GAPDH at each time-point. Heparanase activation is significantly higher in monolayers treated with DENV2 NS1 than in WNV-treated and untreated controls from 0.5–12 hpt (p<0.0001). **(E)** Co-staining of human heparanase I (red) and syndecan-1 (green) in HPMEC treated with DENV2 NS1 (5 μg/ml) after 30 min, 1 and 3 hpt. Manders’ Overlapping Coefficient (MOC) value for the overlapping fraction (merge) is listed to the right of each time point.

As a result of the dynamic equilibrium between biosynthesis and shedding of various HSPG components, perturbation of the EGL upon shearing stress or increased enzymatic activity (i.e., metalloproteinases or heparanase) results in the alteration of distinct EGL functions, including vascular permeability [6, 17]. Heparanase, an endo-β-D-glucuronidase that cleaves GAGs such as HS, is involved in structural remodeling of the ECM and EGL [26, 27]. Analyses of the expression/activation of human heparanase in HPMEC demonstrated that DENV2 NS1 increases the expression of heparanase starting 30 min post-treatment, with a maximum peak expression detected at 6 hpt (Fig 4A and 4B). Heparanase levels induced by DENV2 NS1 were significantly greater than expression levels in untreated control and WNV NS1-treated monolayers. Human heparanase is produced as an inactive precursor (65 kDa) whose activation involves excision of an internal linker segment, yielding the active heterodimer composed of 8 and 50 kDa subunits [28]. Along with the augmented expression of heparanase, increased proteolytic processing of pro-heparanase into an active form (~50 kDa) was detected in HPMEC stimulated with DENV2 NS1 to a much greater degree than WNV NS1-treated and untreated controls (Fig 4C and 4D). Increased enzymatic activity of heparanase has been shown to enhance remodeling of the EGL and ECM, particularly by increasing levels of soluble syndecan-1 on endothelial cells [29, 30]. Immunolocalization of heparanase and syndecan-1 in DENV2 NS1-treated HPMEC showed a temporal pattern of expression and co-localization on the surface of endothelial monolayers (Fig 4E), suggesting that heparanase may induce increased shedding of syndecan-1 in DENV2 NS1-exposed endothelial cells.

**Fig 5:**
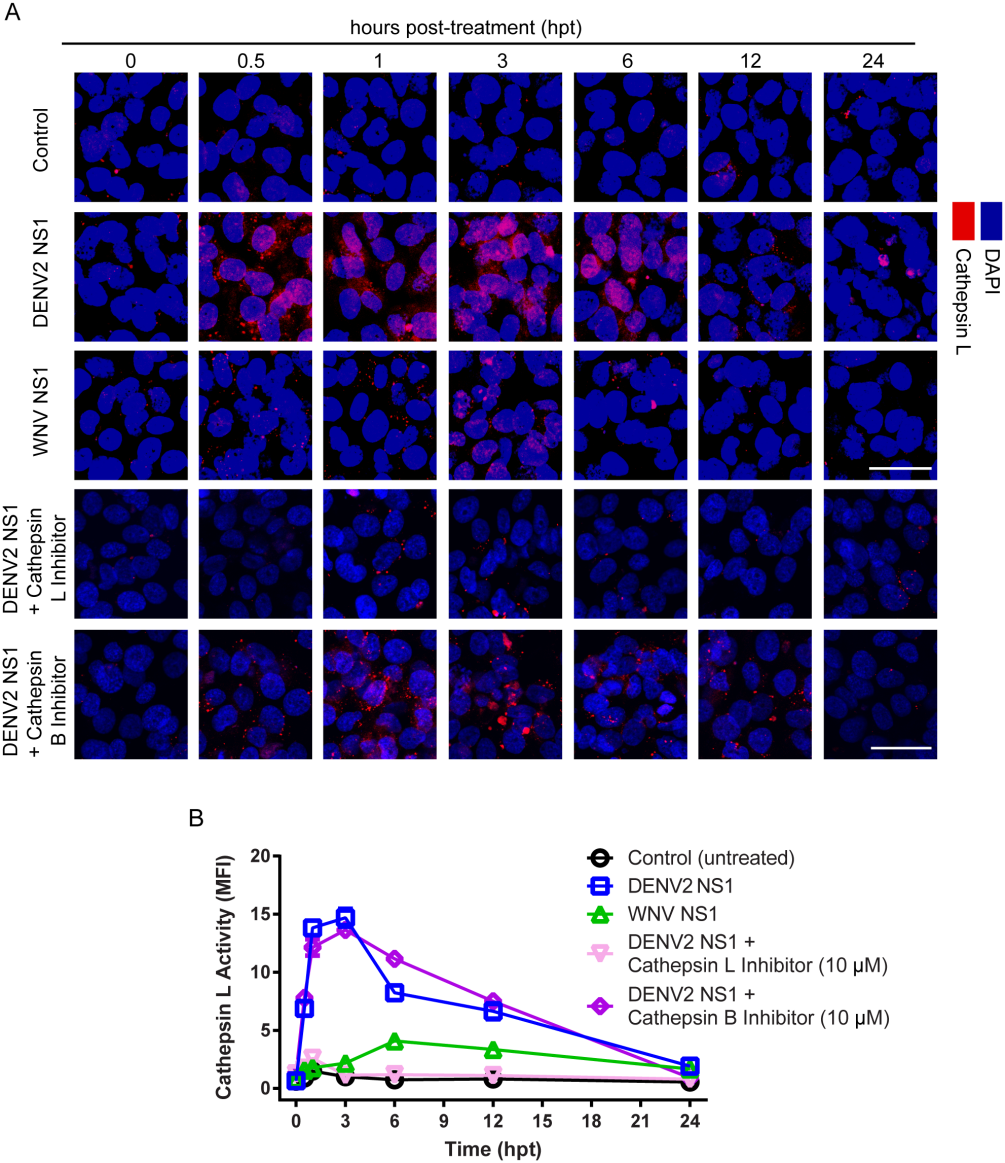
DENV2 NS1 increases the activity of cathepsin L protease. **(A)** Cathepsin L proteolytic activity (Magic Red assay, in red) in HPMEC monolayers over time (hpt) after treatment with DENV2 or WNV NS1 (5 μg/ml) and cathepsin L or B inhibitors (10 μM). Nuclei are stained with *Hoechst* (blue). Untreated cells were used as control for basal cathepsin L expression. Images are representative of three individual experiments (20X). Scale bars, 10 μM. A trace of the MFI for DENV2 NS1 of one representative field is shown below each image. **(B)** Quantification of MFI in (A) from three independent experiments. Staining for cathepsin L activity is significantly higher in monolayers treated with DENV2 NS1 than in WNV-treated and untreated controls at 0.5–12 hpt (p<0.0001). Staining for cathepsin L is significantly lower in monolayers treated with DENV2 NS1 and cathepsin L inhibitor compared to monolayers treated with DENV2 NS1 alone at 0.5–12 hpt (p<0.0001). Error bars indicate SEM throughout.

Activation of heparanase occurs after proteolytic processing by cathepsin L, a ubiquitously expressed endosomal/lysosomal cysteine endopeptidase that is involved in degradation of the ECM [31, 32]. Assessment of cathepsin L activity levels demonstrated that DENV2 NS1 increases the proteolytic activity of intracellular cathepsin L in a time-dependent manner (30 min-12 hpt) in cultured HPMEC to a significantly greater degree than WNV NS1 (Fig 5A, rows 2 and 3 and Fig 5B). This same effect was observed in both HUVEC and HMEC-1 exposed to DENV2 NS1 after 3 and 6 h (S5A and S5B Fig). The activation of cathepsin L by DENV2 NS1 was blocked by a cathepsin L inhibitor but not a cathepsin B inhibitor (Fig 5A, rows 4 and 5 and Fig 5B).

### Heparanase and cathepsin L inhibition blocks DENV2 NS1-triggered EGL disruption and endothelial hyperpermeability

**Fig 6:**
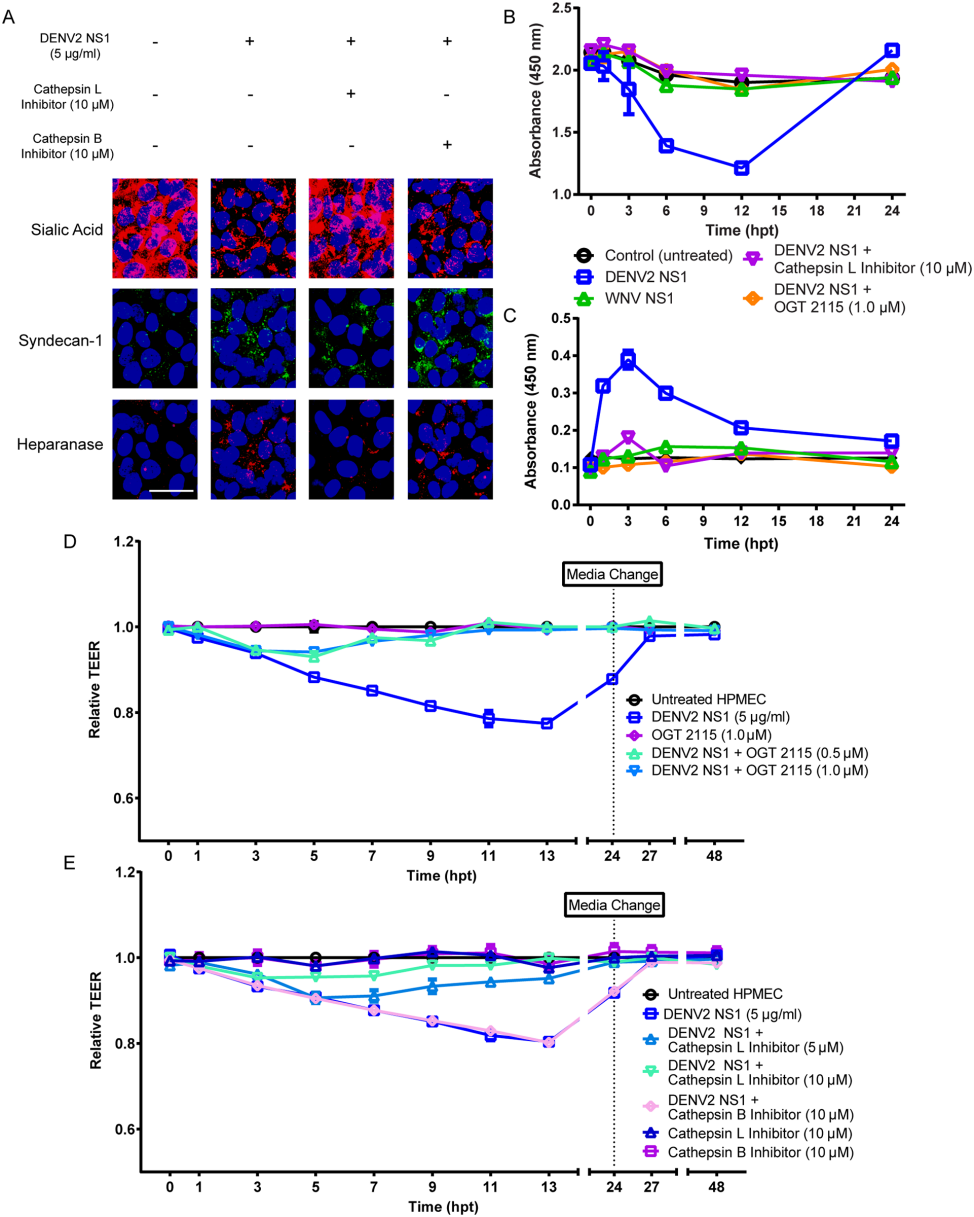
DENV2 NS1-mediated endothelial dysfunction is blocked by inhibition of heparanase activation and cathepsin L activity. **(A)** Effect of cathepsin L inhibitor (10 μM) on DENV2 NS1-mediated disruption of the EGL components Sia (red, upper panels) and syndecan-1 (green, middle panels), and on DENV2 NS1-induced increase of heparanase expression (red, lower panels). Nuclei stained with *Hoechst* (blue). Untreated cells were used as a control for basal expression of EGL components and heparanase. Cathepsin B inhibitor (10 μM) is used as a negative control. Images are representative of three individual experiments (20X). Scale bar, 10 μM. **(B)** Effect of cathepsin L inhibitor and OGT 2115 (human heparanase I inhibitor) on DENV2 NS1-induced release of Sia into the supernatant, as measured by ELISA from three independent experiments. Shedding of Sia is significantly different in monolayers treated with DENV2 NS1 and cathepsin L inhibitor or OGT 2115 than in monolayers treated with DENV2 NS1 alone at 6 and 12 hpt (p<0.0001). **(C)** Effect of cathepsin L inhibitor on DENV2 NS1-induced shedding of syndecan-1, as measured by ELISA from three independent experiments. Shedding of syndecan-1 is significantly lower in monolayers treated with DENV2 NS1 and cathepsin L inhibitor compared to monolayers treated with DENV2 NS1 alone at 1–12 hpt (p<0.0001). **(D)** Effect of OGT 2115 (heparanase I inhibitor) on DENV2 NS1-triggered endothelial hyperpermeability (TEER) in HPMEC monolayers. TEER values of monolayers treated with DENV2 NS1 and OGT 2115 (0.5, 1.0 μM) are significantly different than values of monolayers treated with DENV2 NS1 alone (p<0.0001). **(E)** Effect of cathepsin L inhibitor on DENV2 NS1-induced endothelial hyperpermeability (TEER) in HPMEC monolayers. TEER values of monolayers treated with DENV2 NS1 and cathepsin L inhibitor (5, 10 μM) are significantly different than values of monolayers treated with DENV2 NS1 alone (p<0.0001). In (C) and (D), relative TEER values from three independent experiments performed in duplicate are plotted at indicated time points. In (A) and (C), Cathepsin B inhibitor (CA704, 10 μM) was included as a control for specific cysteine protease inhibition. Error bars indicate SEM throughout.

To confirm the role of cathepsin L in DENV2 NS1-mediated disruption of the EGL, a cathepsin L inhibitor (10 μM), alongside a cathepsin B inhibitor as a control for specificity, was tested in HPMEC monolayers. Alterations of the HPMEC EGL induced by DENV2 NS1, including degradation of sialic acid, shedding of syndecan-1, and increased expression of heparanase, were prevented in the presence of cathepsin L but not cathepsin B inhibitors (Fig 6A). Next, the effect of blocking cathepsin L and/or heparanase, using cathepsin L inhibitor and the heparanase inhibitor OGT 2115 (1.0 μM) [33], respectively, on syndecan-1 and Sia shedding in supernatants of NS1-treated HPMEC was examined by ELISA. The decrease in Sia as well as the increase in syndecan-1 observed in HPMEC supernatants in response to NS1 treatment were both reversed by cathepsin L and/or heparanase inhibitors (Fig 6B and 6C). Both OGT 2115 and cathepsin L inhibitor were used at concentrations that do not affect endothelial cell viability as determined by CellTox Green Cytotoxicity Assay (Promega).

Finally, to characterize the role of endothelial heparanase and cathepsin L in DENV2 NS1-mediated endothelial permeability, cathepsin L inhibitor and OGT 2115 were tested in HPMEC monolayers treated with DENV2 NS1. OGT 2115 induced substantial protection against DENV2 NS1-induced hyperpermeability in HPMEC at 3–7 hpt (Fig 6D), as measured by TEER. HPMEC monolayers exposed to cathepsin L inhibitor were also protected in a dose-dependent manner from DENV2 NS1-induced endothelial hyperpermeability (Fig 6E). In contrast, DENV2 NS1 still increased permeability of HPMEC monolayers in the presence of a cathepsin B-specific inhibitor (Fig 6E). Further, the use of an inhibitor cocktail containing DANA (50 μg/ml), OGT 2115 (1.0 μM), and cathepsin L inhibitor (10 μM) completely prevented DENV2 NS1-induced endothelial hyperpermeability in HPMEC (S6 Fig). Together, these data demonstrate the functional significance of the cathepsin L-heparanase pathway, in that the inhibition of either enzyme prevented both the disruption of the EGL and the hyperpermeability of HPMEC triggered by DENV2 but not WNV NS1.

### TLR4 plays a role in disruption of sialic acid but in not activation of the cathepsin L-heparanase pathway

Because TLR4 has been implicated as a component of DENV2 NS1-induced vascular leak, we investigated the impact of LPS-RS, a TLR4 antagonist, on NS1-induced effects in HPMEC monolayers. We first evaluated Sia expression and found that treatment with DENV2 NS1 in the presence of LPS-RS (50 μg/ml) significantly increased the staining of Sia on the surface of HPMEC by 68–98% compared to DENV2 NS1 alone, suggesting that TLR4 is somehow involved in the disruption of Sia in the EGL (S7A and S7C Fig). LPS-RS also increased the surface staining of syndecan-1 by 14–36% (S7B and S7D Fig) but slightly decreased the activity of cathepsin L by 8–19% in HPMEC (S8A and S8C Fig); the expression of heparanase was unaffected (S8B and S8D Fig). However, monolayers treated with LPS-RS and DENV2 NS1 still showed significant differences in syndecan-1 surface staining (5-fold) and cathepsin L activity (10-fold) compared to untreated controls. Overall, these data suggest that TLR4 may play a role in Sia disruption in the EGL of HPMEC but only minimally affects the cathepsin L-heparanase pathway following binding of DENV2 NS1 to HPMEC, as this pathway is still strongly activated even with inhibition of TLR4.

### NS1 from DENV1, 3, and 4 induces effects similar to DENV2 NS1 in endothelial cells

**Fig 7:**
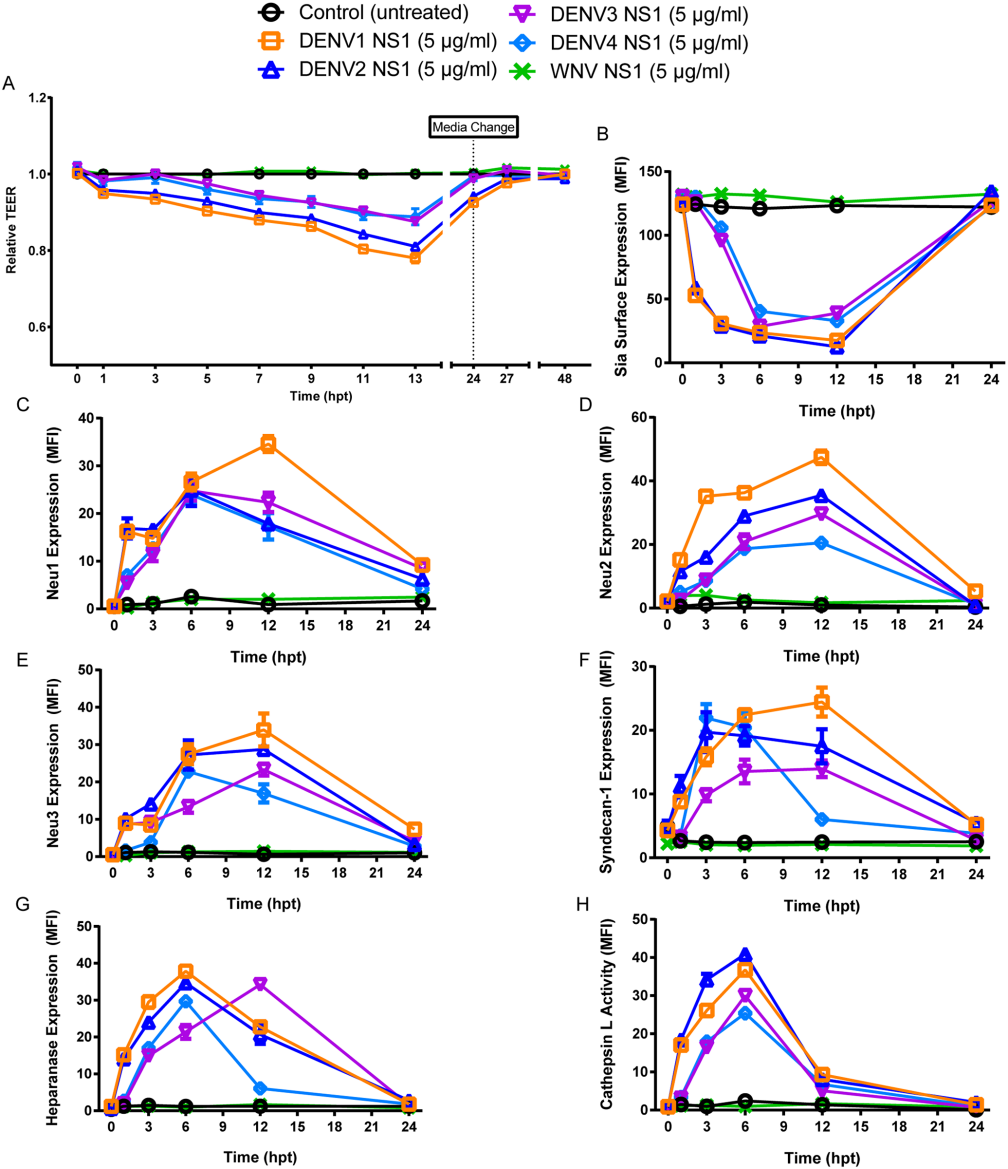
DENV NS1-induced effects on the EGL leading to endothelial hyperpermeability are similar across all four DENV serotypes. **(A)** TEER assay to evaluate the effect of NS1 proteins from DENV1-4 and WNV (5 μg/ml) on HPMEC endothelial permeability. Relative TEER values from three independent experiments performed in duplicate are plotted at the indicated time points. Error bars indicate standard error of the mean (SEM). NS1 from all DENV serotypes induces statistically significant decreases in TEER (p<0.0001), while NS1 from WNV does not. **(B)** Quantification of MFI in S9 Fig from three independent experiments. Sia expression in DENV1 and DENV2 NS1-treated monolayers is significantly different from WNV NS1-treated and untreated controls from 1 to 12 hpt (p<0.0001), and Sia expression in DENV3 and DENV4 NS1-treated monolayers is significantly different from WNV NS1-treated and untreated controls from 3–12 hpt (p<0.0001). **(C)** Quantification of MFI in S10 Fig from three independent experiments. Neu1 expression in DENV1, 2, 3, and 4 NS1-treated monolayers is significantly different from WNV NS1-treated and untreated controls from 1 to 12 hpt (p<0.0001). **(D)** Quantification of MFI in S11 Fig from three independent experiments. Neu2 expression in DENV1, 2, and 4 NS1-treated monolayers is significantly different from WNV NS1-treated and untreated controls from 1 to 12 hpt (p<0.0001), and Neu2 expression in DENV3 NS1-treated monolayers is significantly different from WNV NS1-treated and untreated controls from 3–12 hpt (p<0.0001). **(E)** Quantification of MFI in S12 Fig from three independent experiments. Neu3 expression in DENV1, 2, and 3 NS1-treated monolayers is significantly different from WNV NS1-treated and untreated controls from 1 to 12 hpt (p<0.0001), and Neu3 expression in DENV4 NS1-treated monolayers is significantly different from WNV NS1-treated and untreated controls from 6–12 hpt (p<0.0001). **(F)** Quantification of MFI in S13 Fig from three independent experiments. Syndecan-1 expression in DENV1 and 2 NS1-treated monolayers is significantly different from WNV NS1-treated and untreated controls from 1 to 12 hpt (p<0.0001); Syndecan-1 expression in DENV3 NS1-treated monolayers is significantly different from WNV NS1-treated and untreated controls from 3–12 hpt (p<0.0001); Syndecan-1 expression in DENV4 NS1-treated monolayers is significantly different from WNV NS1-treated and untreated controls from 3–6 hpt (p<0.0001). **(G)** Quantification of MFI in S14 Fig from three independent experiments. Heparanase expression in DENV1 and 2 NS1-treated monolayers is significantly different from WNV NS1-treated and untreated controls from 1 to 12 hpt (p<0.0001), and heparanase expression in DENV3 and 4 NS1-treated monolayers is significantly different from WNV NS1-treated and untreated controls from 3–12 hpt (p<0.0001). **(H)** Quantification of MFI in S15 Fig from three independent experiments. Cathepsin L activity in DENV1, 2, 3, and 4 NS1-treated monolayers is significantly different from WNV NS1-treated and untreated controls from 1 to 12 hpt (p<0.0001).

To determine whether the effects observed in endothelial cells were specific to DENV2 NS1, NS1 from DENV1, 3, and 4 was evaluated using the same experimental setup as previously described. As we have shown previously [9], endothelial permeability of HPMEC was significantly increased following addition of DENV1-4 NS1, as measured by TEER (Fig 7A). Staining for Sia on the surface of HPMEC was significantly decreased 1–12 hpt after treatment with DENV1 and 2 NS1 and 2–12 hpt after treatment with DENV3 and 4 NS1 (Figs 7B and S9). Increased expression of Neu1 was observed in HPMEC monolayers 1–12 hpt following treatment with DENV1-4 NS1 when compared to untreated controls and WNV NS1-treated cells (Figs 7C and S10). Expression of Neu2 was significantly increased 1–12 hpt following treatment with NS1 from DENV1, 2, and 4 and 3–12 hpt following treatment with DENV3 NS1 when compared to untreated and WNV-treated HPMEC (Figs 7D and S11). Neu3 expression was similarly increased 1–12 hpt following treatment with DENV1, 2, and 3 NS1 and 6–12 hpt following treatment with DENV4 NS1 when compared to untreated and WNV NS1-treated controls (Figs 7E and S12). Significantly increased staining of syndecan-1 on the surface of HPMEC monolayers was observed following DENV1-4 NS1 treatment, although the kinetics varied depending on serotype (DENV1,2–1–12 hpt; DENV3–3–12 hpt; DENV4–3–6 hpt) (Figs 7F and S13). Expression of heparanase was also significantly increased following treatment with NS1 from all four DENV serotypes, though the effect was slightly delayed in DENV3-4 (3–12 hpt) compared to DENV1-2 (1–12 hpt) (Figs 7G and S14). Further, cathepsin L activity was significantly increased from 1–12 hpt following treatment with DENV1-4 NS1 (Figs 7H and S15). Taken together, these data demonstrate that NS1 from all four DENV serotypes induces hyperpermeability in endothelial cells using similar molecular mechanisms.

## Discussion

**Fig 8:**
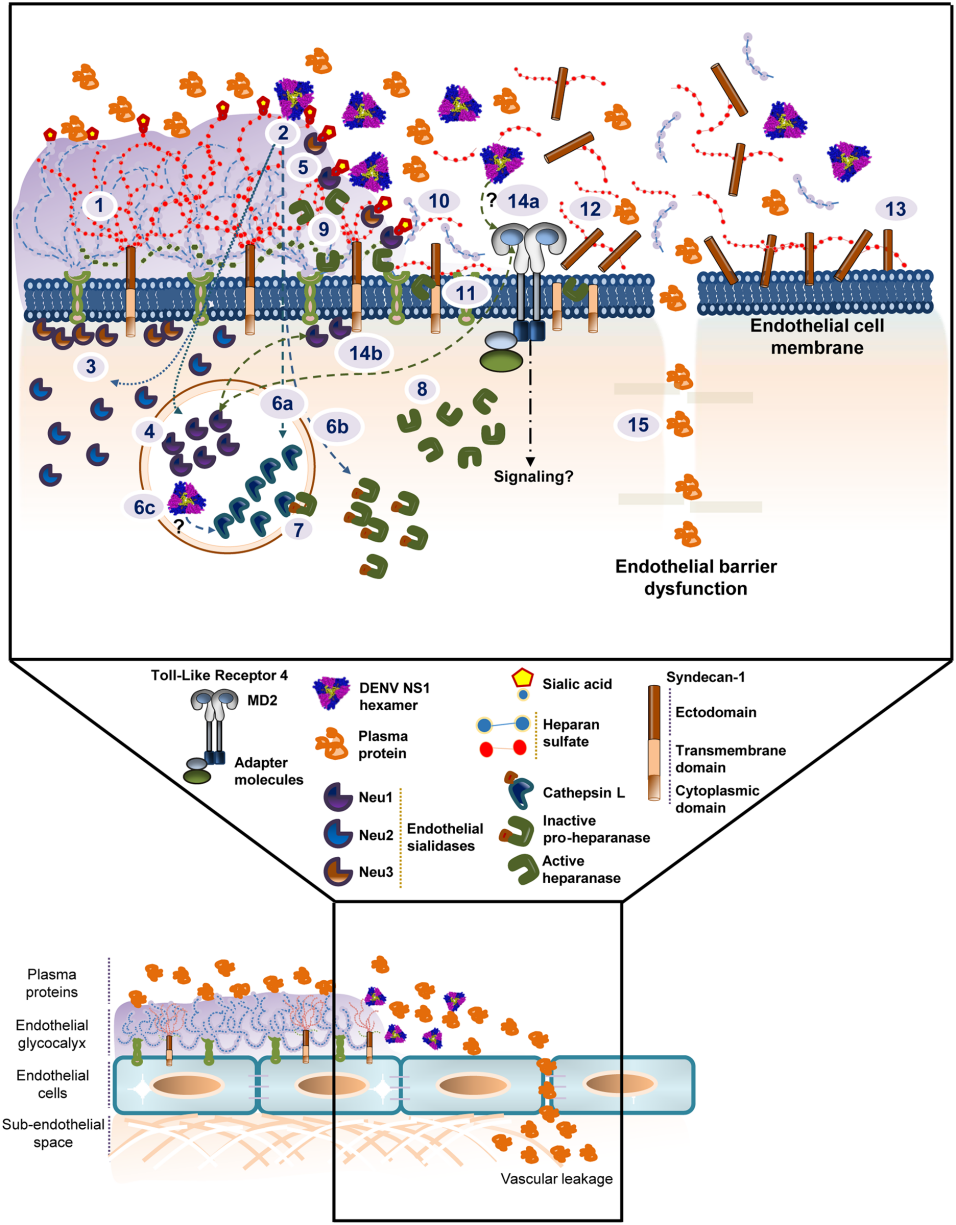
Model of DENV NS1-induced endothelial hyperpermeability of human pulmonary microvascular endothelial cells (HPMEC). **(1)** The endothelial glycocalyx layer (EGL), a network of membrane-bound proteoglycans and glycoproteins, lines the endothelium on the luminal side. Both endothelium- and plasma-derived soluble molecules interact with this mesh. **(2)** Hexameric NS1 protein secreted from flavivirus-infected cells binds to the surface of uninfected cells, including human pulmonary microvascular endothelial cells (HPMEC), upregulating the expression of lysosomal, cytosolic, and cell membrane endothelial sialidases **(3,4)** that translocate to the cell membrane, initiating trimming of terminal sialic acid residues expressed on EGL **(5)**. In addition, DENV NS1 enhances the expression of the inactive precursor of the endoglycosidase (pro-heparanase) **(6a)** and the activity of the lysosomal cysteine protease cathepsin L **(6b)**, potentially through internalization of NS1 **(6c)**. Cathepsin L processes pro-heparanase into an active/mature form **(7,8)**, leading to cleavage of heparan sulfate chains on the EGL **(9,10)**. This results in shedding of syndecan-1, a main component of the EGL **(11,12)**, and its accumulation after binding back to the cell surface **(13)**. Additionally, DENV NS1 may trigger TLR4 signaling **(14a)**, leading to the translocation of Neu1 to the cell membrane and further disruption of sialic acid in the EGL **(14b)**. Together, these processes lead to EGL disruption on the surface of endothelial cells, resulting in endothelial barrier dysfunction and fluid extravasation (hyperpermeability) that occurs in severe dengue disease **(15)**.

Secondary DENV infection with a serotype different from primary infection is considered an epidemiological risk factor for severe disease. Immune responses after primary DENV infection lead to protective immunity against homologous re-infection but may either protect against or cause increased disease severity in a subsequent DENV infection with a different serotype [34]. The latter is thought to be mediated by serotype cross-reactive T cells or antibody-dependent enhancement that triggers an exaggerated and skewed immune response to a previously infecting serotype, resulting in a “cytokine storm”, including TNF-α and IL-6, that leads to endothelial permeability and vascular leak [7]. New evidence has demonstrated the ability of DENV NS1 to directly induce release of vasoactive cytokines via TLR4 stimulation of PBMCs, leading to the disruption of endothelial barrier function *in vitro* and increased vascular leakage *in vivo* [9, 10]. However, NS1-mediated mechanisms specific to the endothelial barrier itself have yet to be defined. Here, we show that binding of DENV NS1 to endothelial cells triggers endothelial barrier dysfunction through alterations to the EGL. DENV NS1 induces the degradation of Sia, a major constituent of the EGL, an effect that is mediated by cellular sialidases. Further, DENV NS1 increases the activity of cathepsin L, which subsequently increases expression and activation of heparanase in endothelial cells, leading to shedding of heparan sulfate proteoglycans from the EGL, thus altering its integrity. Inhibition of sialidases or the cathepsin L-heparanase pathway prevents DENV NS1-mediated disruption of the EGL as well as endothelial hyperpermeability. These results were observed during treatment with amounts of DENV NS1 similar to levels reported in DHF/DSS patients [14, 15] and suggest a novel mechanism whereby soluble NS1 directly interacts with endothelial cells, inducing the activation of endothelial cell-intrinsic pathways that lead to hyperpermeability. A model summarizing these findings is shown in Fig 8.

Endothelial cells are the most important cellular component of the vasculature, separating blood from underlying tissue [35]. In severe dengue disease, plasma leakage occurs in multiple organs around the time of defervescence; however, profuse accumulation of fluids usually takes place in organs such as the lung, where pleural effusion can lead to respiratory distress and shock [3]. Secreted hexameric DENV NS1 has been shown to bind to the surface of cultured human microvascular endothelial cells [11], and aortic and umbilical vein endothelial cells *in vitro* and lung and liver tissues *in vivo* can act as targets for NS1 binding [11]. *In vitro*, we had shown that NS1 from all four DENV serotypes triggers increased permeability of HPMEC monolayers [9]. Our results here demonstrate that NS1 from DENV2 but not from WNV, a closely related flavivirus, binds in a dose-dependent fashion to the surface of HPMEC, and this binding pattern is reflected in the dose-dependent decrease of TEER following the addition of NS1 from DENV2. Similar results were obtained when endothelial cells from different tissues such as HUVEC (umbilical cord) and HMEC-1 (dermis) were exposed to DENV and WNV NS1 proteins [9]. These results support previous observations where inoculation of DENV NS1 alone increased vascular leakage *in vivo* [9] and also suggest that DENV NS1 can modulate endothelial barrier function in different microvascular beds and organs, thereby contributing to the systemic vascular leakage observed in patients experiencing severe dengue disease. Following treatment with DENV NS1, we observe a time-dependent but transient increase in endothelial permeability, and endothelial monolayers recover normal barrier function by 24 hpt, potentially due to loss of NS1 from culture medium as a result of passage to the basolateral compartment, internalization, or degradation. Our *in vitro* model utilizes a single administration of NS1, whereas an acute DENV infection in humans results in continuous production of NS1 from infected cells until the virus is cleared. Following viral clearance, NS1 levels decrease, vascular leakage subsides, and patients recover, reflecting our observations that endothelial hyperpermeability is reversed as NS1 stimulus is lost.

Over the last several decades, the EGL has emerged as a potential regulator of vascular permeability [6]. The negative charge provided by glycoproteins bearing terminal monosaccharides, such as Sia residues, and proteoglycans bearing GAGs, such as HS, chondroitin sulfate, and hyaluronic acid [5, 36, 37], contributes to the barrier function of the EGL. To examine the integrity of the EGL, we initially evaluated the distribution of Sia by staining with the lectin WGA and found that NS1 from all four DENV serotypes significantly reduces Sia staining on the surface of HPMEC monolayers. This effect does not occur in the presence of WNV NS1. Due to its prominent position as the outermost monosaccharide unit on the glycan chains of glycolipids and glycoproteins in the EGL as well as its negative charge, Sia is involved in a variety of functions, including regulation of vascular permeability [5, 16, 36–39]. Therefore, removal of Sia from the EGL may result in reduction of the net negative charge and hydrophilicity of the endothelial surface [5, 36]. Accordingly, disruption of Sia on the EGL may play a key role in DENV NS1-induced endothelial barrier dysfunction observed in HPMEC.

DENV NS1 has been reported to bind to uninfected cells primarily via interactions with HS and chondroitin sulfate E [11]. In this study, soluble NS1 showed a similar binding pattern to that of the lectin WGA on HPMEC. WGA has been shown to bind to Sia and to *N*-acetylglucosamine [40]. However, the striking reduction of WGA binding to HPMEC after neuraminidase treatment suggests that Sia is a major constituent of the glycan moieties present on HPMEC, consistent with previously reports for other microvascular beds [17], and is thus a major interaction partner for DENV NS1.

In eukaryotic systems, Sia can be metabolized via enzymatic release or degradation by sialidases/neuraminidases or Sia-specific pyruvate lyases [16]. As such, reduced Sia expression in HPMEC exposed to DENV NS1 may be a consequence of enzymatic trimming by endothelial sialidases. Our data demonstrate that free Sia levels in conditioned media were significantly reduced in DENV NS1-treated HPMEC compared to untreated or WNV NS1-treated monolayers, indicating that DENV NS1 may trigger degradation rather than release of Sia from the cell surface. Furthermore, no endothelial sialidase activity was found in supernatant collected from DENV NS1-treated HPMEC, indicating that Sia on endothelial cells is removed by the action of specific membrane-associated sialidases and/or metabolized by intracellular lyases. Analyses by confocal microscopy identified that Neu1, Neu2, and Neu3 sialidases were selectively upregulated in HPMEC in the presence of all DENV NS1 proteins but not WNV NS1. Neu1 is mainly localized in lysosomes but is also capable of translocation to the cell surface [41]. Neu2, also known as the soluble sialidase, is a cytosolic enzyme that cleaves a variety of substrates, including oligosaccharides, glycoproteins, and gangliosides (Sia-containing glycolipids) [42]. Neu3 is found on the cell membrane, acting specifically on the sialic acids of gangliosides [39]. Thus, increased expression of endothelial sialidases triggered by DENV NS1 may lead to trimming of Sia on the surface of HPMEC, resulting in initial degradation of the EGL and increased endothelial permeability. Additionally, we found that treatment of HPMEC monolayers with Zanamivir or DANA, influenza neuraminidase inhibitors that have also been shown to significantly inhibit human sialidases [19], substantially protects endothelial monolayers from DENV NS1-induced hyperpermeability. These data suggest that removal of Sia from the EGL by human sialidases contributes to increased permeability of human endothelial cell monolayers following binding of DENV NS1.

In vertebrates, mammalian sialidases and their target substrates have been implicated in crucial biological processes, including the regulation of cell proliferation/differentiation, clearance of plasma proteins, control of cell adhesion, metabolism of gangliosides and glycoproteins, immunocyte function, and modification of receptors [39]. More recently, a novel role for Neu1 in controlling the activation of TLR4 signaling pathways was described [43, 44]. Briefly, Neu1 activity has been shown to influence receptor desialylation and disruption of TLR4:Siglec-E interaction, which subsequently activates TLR4 signaling, leading to the production of nitric oxide and pro-inflammatory cytokines in dendritic and macrophage cells [43–47]. Further, TLR4 signaling has been shown to be required for translocation of Neu1 to the cell membrane [45]. Thus, DENV NS1 stimulation of Neu1 may lead to TLR4 signaling, in turn contributing to the translocation of Neu1 to the cell membrane and subsequent disruption of Sia in the EGL of HPMEC. Interestingly, we found that when HPMEC monolayers are treated with LPS-RS, a TLR4 antagonist that binds MD-2 in the TLR4 complex, DENV NS1-induced disruption of Sia is significantly decreased. This suggests that treatment with LPS-RS may prevent TLR4 signaling and ensuing translocation of Neu1 to the cell membrane, thereby partially preventing the disruption of Sia that occurs after treatment with DENV NS1 alone.

In addition to Sia residues, cell surface proteoglycans and their associated GAG side chains help to preserve the stability and function of the EGL. Transmembrane syndecans, membrane-bound glypicans, and basement matrix-associated perlecans are the three major protein core families of HSPGs found on endothelial cells [6, 23]. Structurally, syndecans are composed of an N-terminal signal peptide, an extracellular domain containing several consensus sequences for GAG attachment, a single transmembrane domain, and a short C-terminal cytoplasmic domain [48]. Syndecan ectodomains can be shed intact by proteolytic cleavage of their core proteins [49, 50]. Due to its HS chains, syndecan-1 can function as a co-receptor on the cell surface and also as a soluble HSPG that binds to a wide variety of extracellular ligands, including matrix proteins, cytokines, and chemokines. In this study, a specific immunoassay to detect soluble syndecan-1 from conditioned HPMEC media demonstrated that DENV NS1 induces enhanced shedding of the syndecan-1 ectodomain from the EGL. Since the *in vitro* HPMEC monolayer system is static, this shedding may lead to increased deposition and accumulation of syndecan-1 on the surface of HPMEC, thereby explaining the increased signal for syndecan-1 detected by confocal microscopy. The shedding of syndecan-1 can then result in increased stimulation of inflammatory signaling pathways in the endothelium. Elevated levels of syndecan-1 ectodomains have been implicated in adhesion, migration, cytoskeleton organization, cell differentiation, and vascular permeability [48]. Here, we showed that recombinant syndecan-1 increases permeability when added to HPMEC, suggesting that altered expression, distribution, and release of HSPGs (e.g., syndecan-1) from the surface of HPMEC after stimulation with DENV NS1 may result in the activation of inflammatory processes that contribute to endothelial barrier dysfunction.

Accelerated shedding of syndecan-1 has been shown to result from direct proteolytic cleavage by matrix metalloproteinases (MMP) [49, 50]. However, syndecan shedding has also been found to be enhanced by enzymatic degradation of HS chains, indicating that non-MMP mechanisms are also involved in this process [29, 51]. Heparanase is a β-D-endoglucuronidase that cleaves HS, facilitating degradation of the EGL and the ECM and resulting in release of proteoglycans bearing HS, such as syndecan-1 [27, 29, 30]. Remodeling of the EGL and ECM by heparanase is important for various physiological and pathological processes, including inflammation, wound healing, tumor angiogenesis, and metastasis [52]. Human pro-heparanase is produced as an inactive precursor protein (~543 amino acids) whose activation involves excision of an internal linker segment (Ser110–Gln157), yielding the active heterodimer composed of 8 and 50 kDa subunits [27]. Processing and activation of pro-heparanase requires cathepsin L, a papain-like lysosomal cysteine proteinase that is ubiquitously expressed in human tissues and is involved in normal cellular protein degradation and turnover [32]. Here, analyses of HPMEC monolayers by confocal microscopy demonstrated an increase of heparanase staining and cathepsin L protease activity, detected as early as 30 min after endothelial cell stimulation with DENV but not WNV NS1. Increased expression of the active form of heparanase (~50 kDa) was also shown, indicating that the DENV NS1-induced endothelial hyperpermeability may result from enhanced processing and activation of heparanase by intracellularly expressed cathepsin L. Cathepsin L and heparanase may thus play a critical role in NS1-induced disruption of HS and HSPG components of the EGL, such as syndecan-1. Though MMPs are primarily responsible for the homeostasis of the ECM, cysteine proteases can significantly contribute to its destruction under disease conditions [32]. Increased cathepsin L activity has been found to promote disease pathogenesis by creating an inflammatory environment associated with degradation of the ECM in cardiovascular disease, cancer, and rheumatoid arthritis [32]. Further, heparanase is upregulated in numerous human diseases such as cancer, diabetes, renal disease, and Alzheimer disease [52, 53]. Therefore, overexpression of endothelial heparanase and its increased processing by lysosomal cathepsin L may constitute a key component of the intrinsic endothelial mechanisms initially triggered by DENV NS1, leading to the disruption of EGL integrity that contributes endothelial barrier dysfunction in endothelial cell monolayers.

The mechanism by which DENV NS1 induces increased activity of cathepsin L is still unclear. Cathepsins are lysosomal cysteine proteases mainly responsible for the remodeling of the extracellular matrix (ECM) [32]. They are optimally active at a slightly acidic pH; however, the mechanism of their activation is not fully understood. We have obtained preliminary results that indicate that DENV NS1 is not only able to interact with the surface of the endothelium but also may be internalized and subsequently transported through endothelial monolayers via unidentified endocytic pathways, leading to its accumulation in basolateral compartments. It is possible that this NS1 internalization process leads to the activation of cathepsin L in endosomes of HPMEC, thus contributing to subsequent degradation of the ECM and NS1-induced endothelial hyperpermeability. Alternatively, it is possible that cathepsin L is activated via a sequence of molecular signals following DENV NS1 binding to the surface of endothelial cells.

Our data suggest that DENV NS1 induces endothelial hyperpermeability through significant disruption of the EGL, a phenomenon that may be primarily regulated by the activation the cathepsin L-heparanase pathway. This conclusion was further tested through the use of specific inhibitors of both heparanase (OGT 2115) and cathepsin L (cathepsin L inhibitor). Endothelial hyperpermeability induced by DENV NS1 in HPMEC monolayers was significantly reversed in the presence of OGT 2115 and cathepsin L inhibitor and was completely reversed in the presence of an inhibitor cocktail containing DANA, OGT 2115, and cathepsin L inhibitor. Further, disruption of Sia, increased surface staining of syndecan-1, and increased activation of heparanase were prevented after inhibition of cathepsin L activity. Notably, when an inhibitor for cathepsin B, a related cysteine protease [32], was used, neither increased endothelial permeability nor EGL disruption was inhibited in DENV NS1-treated HPMEC monolayers. These data support our conclusion that activation of EGL remodeling pathways play a significant role in the endothelial barrier dysfunction induced by DENV NS1.

This work provides insight into endothelial cell-intrinsic mechanisms that contribute to endothelial hyperpermeability triggered by DENV NS1 protein. We have identified multiple pathways that were previously not known to play a role in severe DENV disease, including disruption of the EGL through endothelial sialidases and the cathepsin L-heparanase pathway. The full story is still incomplete, as the precise timing and signaling cascades remain to be defined, and future work will need to further elucidate these kinetics. More comprehensive studies are underway to understand the relative contribution of these endothelial-intrinsic mechanisms in the context of dengue disease, as other factors, including vasoactive cytokines triggered by NS1 [9, 10] and immunopathogenic mechanisms [54], are known to play an important role in DHF/DSS. Overall, these findings add to the novel functions of DENV NS1 and the discovery of new potential pathways contributing to endothelial dysfunction and vascular leak during severe dengue disease, and they may contribute to future advancements in dengue treatment and diagnostics.

## Materials and Methods

### Cell culture

The human pulmonary microvascular endothelial cell line HPMEC-ST1.6R was kindly donated by Dr. J.C. Kirkpatrick (Institute of Pathology, Johannes Gutenberg University, Germany) and propagated (passages 5–8) and maintained at 37°C in humidified air with 5% CO_2_ in endothelial cell basal medium-2 supplemented with growth factors, antibiotics, and fetal bovine serum as per the manufacturer’s specifications (Clonetics, Lonza). The human dermal microvascular endothelial cell line HMEC-1 was kindly donated by Dr. M. Welch (University of California, Berkeley) and propagated (passages 20–25) and maintained at 37°C in humidified air with 5% CO_2_ in MCDB 131 medium (Sigma) supplemented with 0.2% Epidermal Growth Factor and 0.4% hydrocortisone. Human Umbilical Vein microvascular endothelial cells (HUVEC) were grown as previously described [9].

### Antibodies, recombinant proteins, and inhibitors

For staining of EGL components, the following monoclonal antibodies (mAbs) and lectins were used: Wheat germ agglutinin (WGA) lectin conjugated to Alexa 647 (WGA-A647, Molecular Probes) to stain N-acetyl neuraminic acid (Sia); anti-human heparanase 1 (HPA1, Santa Cruz Biotech); anti-human cathepsin L (eBioscience); anti-heparan sulfate proteoglycan 2 for perlecan (Abcam), anti-human CD138 for syndecan-1 (eBioscience); Neu1 antibody (H-300): sc-32936 (Santa Cruz Biotech); Neu2 antibody PA5-35114 (Thermo Scientific); Ganglioside sialidase antibody (N-18): sc-55826 for Neu3 (Santa Cruz Biotech). Recombinant NS1 proteins from DENV1 (strain Nauru/Western Pacific/1974), DENV2 (strain Thailand/16681/84), DENV3 (strain Sri Lanka D3/H/IMTSSA-SRI/2000/1266), DENV4 (strain Dominica/814669/1981) and WNV (New York NY99 strain) used in all experiments were produced by Native Antigen (Oxfordshire, United Kingdom) in HEK 293 cells and were shown to be >95% pure and oligomeric, as demonstrated by native PAGE and Western blot analyses [9]. In addition, the NS1 proteins were tested and shown to be free of endotoxin contaminants, as determined using the Endpoint Chromogenic Limulus Amebocyte Lysate (LAL) QCL-1000TM kit (Lonza) (<0.1 EU/ml) and as certified by the manufacturer. Recombinant syndecan-1/CD138 used in TEER assays was >95% pure and <1.0 EU/μg endotoxin by LAL assay according to the manufacturer’s indications (R&D Systems). Recombinant neuraminidase from *Clostridium perfringens* (*C*. *welchii*) was obtained from Sigma. Selective inhibitors of human heparanase (OGT 2115, Tocris), cathepsin L (Cathepsin L inhibitor I, Calbiochem), cathepsin B (CA-074, Tocris Bioscience), neuraminidase (Zanamivir and *N*-Acetyl-2,3-dehydro-2-deoxyneuraminic acid (Sigma)) were used in TEER assays at concentrations that do not affect cell viability. Cell viability was determined by the Promega CellTox Green Cytotoxicity Assay following manufacturer’s instructions.

### Flavivirus NS1 protein binding assay

Confluent HPMEC monolayers grown on gelatin-coated coverslips (0.2%, Sigma) were exposed to different concentrations of DENV2 NS1 (1.25–10 μg/ml) and WNV NS1 (5–10 μg/ml) and incubated for one hour at 37°C. NS1 protein bound to the cell surface was then detected using the anti-NS1 mAb 9NS1 conjugated to Alexa 488 (cross-reactive to WNV and DENV2 NS1; gift from Dr. M.S. Diamond, Washington University in St. Louis) [55] and the anti-NS1 mAb 7E11 conjugated to Alexa 568 (gift from Dr. R. Putnik, Walter Reed Army Institute of Research). For the time course of DENV2 NS1 binding, 5 μg/ml of NS1 was used, and cell monolayers were incubated as described above and fixed (PFA 2%) at 1, 3, 6, 12 and 24 hpt. Images were acquired using a Zeiss LSM 710 AxioObserver-34-channel spectral detector confocal microscope and processed using ImageJ software [56]. A quantification of NS1 protein bound to the cell surface was expressed as mean fluorescence intensity (MFI) compared to untreated cells used as a negative control.

### Trans-Endothelial Electrical Resistance (TEER)

The effect of recombinant NS1 proteins on endothelial permeability was evaluated by measuring TEER [Ohms (Ω)] in HPMEC monolayers grown on a 24-well Transwell polycarbonate membrane system (Transwell permeable support, 0.4 μM, 6.5 mm insert; Corning Inc.) as previously described [9]. Untreated HPMEC grown on Transwell inserts were used as negative untreated controls, and inserts with medium alone were used for blank resistance measurements. Relative TEER represents a ratio of resistance values (Ω) obtained at sequential 2-h time points following the addition of test proteins as follows: (Ω experimental condition—Ω medium alone)/(Ω non-treated endothelial cells– Ω medium alone). After 24 h of treatment, 50% of upper and lower chamber media was replaced by fresh endothelial cell medium. An Epithelial Volt Ohm Meter (EVOM) with “chopstick” electrodes (World Precision Instruments) was used to measure TEER values.

### Fluorescence microscopy

For imaging experiments, HPMEC were grown on coverslips and imaged on a Zeiss LSM 710 Axio Observer inverted fluorescence microscope equipped with a 34-channel spectral detector. Images acquired using the Zen 2010 software (Zeiss) were processed and analyzed with ImageJ software [56]. Cells were counted and MFI values were obtained by using ImageJ cell counter analyses with a viewing area of ~103 μm^2^ (10.28x10.28 μm), which contains roughly 200 cells. For representative pictures, an area of ~1.8 μm^2^ (1.25x1.40 μm) containing ~28–30 cells was used. All RGB images were converted to grayscale, then mean grayscale values and integrated density from selected areas were taken along with adjacent background readings and plotted as mean fluorescence intensity (MFI). To assess the effect of flavivirus NS1 on integrity of the endothelial architecture, the distribution of EGL components was examined on confluent HPMEC monolayers treated with DENV or WNV NS1 proteins (5 μg/ml) and fixed with 2% paraformaldehyde (PFA) and ethanol-methanol (1:1) at different time points (0, 30 min, 1, 3, 6, 12 and 24 hpt). Primary antibodies were incubated overnight at 4°C, and detection was performed using secondary species-specific anti-IgG antibodies conjugated to Alexa fluorophores (488, 568 and 647).

### Western blot

For protein expression, confluent HPMEC monolayers (~1x10^6^ cells/well, 6-well tissue culture-treated plates) were treated with DENV and WNV NS1 proteins (5 μg/ml), and at different time points (0, 30 min, 1, 3, 6, 12 and 24 hpt), cell monolayers were scraped on ice using RIPA lysis buffer (50 mM Tris [pH 7.4], 150 mM NaCl, 1% [v/v] Nonidet-P40, 2 mM EDTA, 0.1% [w/v] SDS, 0.5% Na-deoxycholate and 50 mM NaF) supplemented with complete protease inhibitor cocktail (Roche). After total protein quantification using a bicinchoninic acid (BCA)-based colorimetric assay (Pierce BCA Protein Assay Kit, Thermo Scientific), 10 μg of total protein per sample was boiled and placed in reducing Laemmeli buffer and separated by 4–20% gradient SDS-PAGE. After immunoblotting using specific primary antibodies for syndecan-1, human heparanase, human cathepsin L, and GAPDH (used as housekeeping protein control) and secondary species-specific anti-IgG antibody conjugated to Alexa 680 or Alexa 750, protein detection and quantification was carried out using the Odyssey CLx Infrared Imaging System (LI-COR). Relative densitometry represents a ratio of the values obtained from each experimental protein band over the values obtained from loading controls (GAPDH) after subtracting background from both using Image Studio Lite V 5.2 (LI-COR Biosciences).

### ELISA

ELISAs for human syndecan-1 (CD138), Sia (NANA), and human cathepsin L were performed following the manufacturer’s instructions (Abcam).

### Enzymatic activity assays

Cathepsin L activity in living cells was monitored using the Magic Red Cathepsin L detection kit (Immunochemistry Technologies, Inc.). Briefly, confluent HPMEC monolayers grown on coverslips were exposed to DENV and WNV NS1 proteins (5 μg/ml), and at different time points, a cell membrane-permeant fluorogenic substrate MR-(Phe-Arg)_2_, which contains the cresyl violet (CV) fluorophore branded as Magic Red (MR), was added. Cultured cell monolayers expressing active cathepsin L catalyze the hydrolysis of the two Phe-Arg target sequences, generating a red fluorescent species that can be detected by immunofluorescence microscopy. Magic Red excites at 540–590 nm (590 nm optimal) and emits at >610nm (630 nm optimal). For neuraminidase detection, culture supernatants from NS1-exposed HPMEC monolayers were collected at different time points and processed for neuraminidase activity using the Amplex Red reagent-based assay and fluorescence detection following recommended procedures (Molecular Probes).

### Statistical analysis

Statistical analysis was performed using GraphPad Prism 6 software, and all graphs were generated using Prism 6. Comparison between MFI, ELISA, and densitometry data was conducted using multiple t-tests with a False Discovery Rate of 1%. For TEER experiments, statistical significance was determined using a two-way analysis of variance (ANOVA).

## Supporting Information

S1 Fig Related to Figs 1 and 2C: Binding of DENV2 NS1 to endothelial cells is both a dose- and time-dependent effect. **(A)** Binding of DENV2 NS1 to HPMEC monolayers, examined by confocal microscopy. DENV2 NS1 was stained with a specific monoclonal antibody (9NS1 conjugated to Alexa 488), and Sia was stained with WGA-A647 (red) at indicated time points (hpt). Nuclei stained with *Hoechst* (blue). Images (20X) are representative of three independent experiments. Scale bar, 10 μM. **(B)** Quantification of MFI of DENV2 NS1 staining in **S1B Fig** from three independent experiments. **(C)** DENV2 NS1 disrupts Sia on the EGL of HPMEC in a dose-dependent manner. Quantification of Sia expression on cell surface and DENV2 NS1 binding to HPMEC monolayers in the presence of different concentrations of DENV2 NS1 (1.25, 2.5, 5, and 10 μg/ml) in Fig 2C. Results represent mean fluorescence intensity (MFI) values from three independent experiments. Grey bars represent relative sialic acid expression, calculated by normalizing each condition to untreated control cells (relative sialic acid expression = sialic acid expression in DENV2 NS1-treated monolayers/sialic acid expression in untreated control monolayers). The blue line represents DENV2 NS1 binding.
(TIF)

S2 Fig Related to Fig 1: DENV2 NS1-induced endothelial hyperpermeability is not a result of LPS contamination and occurs in other endothelial cell types. **(A)** TEER assay to evaluate the effect of DENV2 NS1 (5 μg/ml) on HMEC-1 endothelial permeability. Relative TEER values from one independent experiment performed in duplicate are plotted at the indicated time points. Error bars indicate standard error of the mean (SEM). DENV2 induces statistically significant decreases in TEER (p<0.05). **(B)** Effect of polymyxin B on DENV2 NS1-mediated endothelial hyperpermeability (TEER) in HPMEC monolayers. Relative TEER values from three independent experiments performed in duplicate are plotted at indicated time points (hpt). TEER values for monolayers treated with DENV2 NS1 combined with polymyxin B (25 μg/ml) are not significantly different from monolayers treated with DENV2 NS1 alone. Error bars indicate SEM throughout.
(TIF)

S3 Fig Related to Fig 2: The lectin WGA primarily binds to sialic acid on the surface of HPMEC, and DENV2 NS1 disrupts Sia expression on the surface of different endothelial cells. **(A)** Sia expression on HPMEC monolayers after treatment with recombinant neuraminidase from *Clostridium perfringens* (0.5 UI), examined by confocal microscopy. Sia was stained with WGA-A647 (red) at indicated time points (hpt). Untreated cells were used as a control for basal Sia expression. Nuclei stained with *Hoechst* (blue). Images (20X) are representative of three independent experiments. Scale bar, 10 μM. **(B)** Quantification of MFI in **S3A Fig** from three independent experiments. Sia expression in monolayers treated with recombinant neuraminidase is significantly different than in untreated control monolayers (p<0.0001). **(C)** Sia expression on HUVEC monolayers after treatment with DENV2 NS1 (5 μg/ml), examined by confocal microscopy. Sia was stained with WGA-A647 (red) at indicated time points (hpt). Nuclei are stained with *Hoechst* (blue). Untreated cells were used as control for basal Sia expression. Images are representative of one individual experiment (20X). **(D)** Sia expression on HMEC-1 monolayers after treatment with DENV2 NS1 (5 μg/ml), examined by confocal microscopy. Sia was stained with WGA-A647 (red) at indicated time points (hpt). Nuclei are stained with *Hoechst* (blue). Untreated cells were used as control for basal Sia expression. Images are representative of one individual experiment (20X).
(TIF)

S4 Fig Related to Fig 3: DENV2 NS1 increases the surface staining of syndecan-1 and perlecan in the EGL of HPMEC. **(A)** Quantification of syndecan-1 MFI in Fig 3A. Staining is significantly higher with DENV2 NS1 compared to controls at 0.5–12 hpt (p<0.0001). Error bars indicate SEM. **(B)** Expression of perlecan (green) on the surface of HPMEC monolayers over time (hpt) after treatment with DENV2 or WNV NS1 proteins (5 μg/ml), examined by confocal microscopy. Untreated cells were used as a control for basal perlecan expression. Nuclei are stained with *Hoechst* (blue). Images are representative of three individual experiments (20X). Scale bar, 10 μM. A trace of the MFI of one representative field is shown below each image.
(TIF)

S5 Fig Related to Fig 5: DENV2 NS1 increases cathepsin L activity in both HMEC-1 and HUVEC monolayers. **(A)** Cathepsin L proteolytic activity (Magic Red assay, in red) in HUVEC monolayers over time (hpt) after treatment with DENV2 NS1 (5 μg/ml). Nuclei are stained with *Hoechst* (blue). Untreated cells were used as control for basal cathepsin L expression. Images are representative of one individual experiment (20X). **(B)** Cathepsin L proteolytic activity (Magic Red assay, in red) in HMEC-1 monolayers over time (hpt) after treatment with DENV2 NS1 (5 μg/ml). Nuclei are stained with *Hoechst* (blue). Untreated cells were used as control for basal cathepsin L expression. Images are representative of one experiment (20X).
(TIF)

S6 Fig Related to Fig 6: DENV2 NS1-induced endothelial hyperpermeability is blocked by an inhibitor cocktail of DANA, OGT 2115, and cathepsin L inhibitor. Effect of an inhibitor cocktail (DANA, 50 μg/ml; OGT 2115, 1.0 μM; cathepsin L inhibitor, 10 μM) on DENV2 NS1-triggered endothelial hyperpermeability (TEER) in HPMEC monolayers. TEER values of monolayers treated with DENV2 NS1 plus the inhibitor cocktail are significantly different than values of monolayers treated with DENV2 NS1 alone (p<0.0001) and not significant compared to untreated control monolayers.
(TIF)

S7 Fig Related to Fig 6: LPS-RS, an LPS antagonist, partially prevents DENV2 NS1-induced modulation of Sia expression and further increases surface staining of syndecan-1 in the EGL of HPMEC. **(A)** Sia expression on HPMEC monolayers after treatment with DENV2 NS1 (5 μg/ml) and LPS-RS (50 μg/ml), examined by confocal microscopy. Sia was stained with WGA-A647 (red) at indicated time points (hpt). Untreated cells were used as a control for basal Sia expression. Nuclei stained with *Hoechst* (blue). Images (20X) are representative of three independent experiments. Scale bar, 10 μM. **(B)** Staining of syndecan-1 (green) on the surface of HPMEC monolayers over time (hpt) after treatment with DENV2 NS1 (5 μg/ml) and LPS-RS (50 μg/ml), examined by confocal microscopy. Untreated cells were used as a control for basal syndecan-1 expression. Nuclei are stained with *Hoechst* (blue). Images are representative of three individual experiments (20X). Scale bar, 10 μM. **(C)** Quantification of MFI in S12A Fig from three independent experiments. Sia expression in monolayers treated with DENV2 NS1 and LPS-RS is significantly higher than in monolayers treated with only DENV2 NS1 at 1, 3, and 6 hpt (p<0.0001). **(D)** Quantification of MFI in S12B Fig from three independent experiments. Syndecan-1 expression in monolayers treated with DENV2 NS1 and LPS-RS is significantly higher than in monolayers treated with only DENV2 NS1 at 3 and 6 hpt (p<0.0001).
(TIF)

S8 Fig Related to Fig 6: LPS-RS, an LPS antagonist, partially prevents DENV2 NS1-induced increase of cathepsin L activity in HPMEC. **(A)** Cathepsin L proteolytic activity (Magic Red assay, in red) in HPMEC monolayers over time (hpt) after treatment with DENV2 NS1 (5 μg/ml) and LPS-RS, examined by confocal microscopy. Nuclei are stained with *Hoechst* (blue). Untreated cells were used as control for basal cathepsin L expression. Images are representative of three individual experiments (20X). **(B)** Heparanase expression (red) in HPMEC monolayers over time (hpt) after treatment with DENV2 NS1 (5 μg/ml) and LPS-RS, examined by confocal microscopy. Untreated cells were used as a control for basal heparanase expression. Nuclei stained with *Hoechst* (blue). Images are representative of three individual experiments (20X). Scale bar, 10 μM. **(C)** Quantification of MFI in S13B Fig from three independent experiments. Cathepsin L activity in monolayers treated with DENV2 NS1 and LPS-RS is significantly lower than in monolayers treated with only DENV2 NS1 at 3 and 6 hpt (3 hpt, p<0.0001; 6pht, p<0.005). **(D)** Quantification of MFI in S13A Fig from three independent experiments. Heparanase expression in monolayers treated with DENV2 NS1 and LPS-RS is not significantly different than in monolayers treated with only DENV2 NS1.
(TIF)

S9 Fig Related to Fig 7B: NS1 protein from all four DENV serotypes but not WNV modulates the expression of Sia in the EGL of HPMEC. Sia expression on HPMEC monolayers after treatment with NS1 protein from DENV1-4 or WNV (5 μg/ml), examined by confocal microscopy. Sia was stained with WGA-A647 (red) at indicated time points (hpt). Untreated cells were used as a control for basal Sia expression. Nuclei stained with *Hoechst* (blue). Images (20X) are representative of three independent experiments. Scale bar, 10 μM.
(TIF)

S10 Fig Related to Fig 7C: NS1 protein from all four DENV serotypes but not WNV increases the expression of Neu1 in HPMEC. Neu1 expression in HPMEC monolayers after treatment with NS1 protein from DENV1-4 or WNV (5 μg/ml), examined by confocal microscopy. Neu1 was stained with a specific polyclonal antibody (Neu1 antibody (H-300): sc-32936) (green) at indicated time points (hpt). Untreated cells were used as a control for basal Neu1 expression. Nuclei stained with *Hoechst* (blue). Images (20X) are representative of three independent experiments. Scale bar, 10 μM.
(TIF)

S11 Fig Related to Fig 7D: NS1 protein from all four DENV serotypes but not WNV increases the expression of Neu2 in HPMEC. Neu2 expression in HPMEC monolayers after treatment with NS1 protein from DENV1-4 or WNV (5 μg/ml), examined by confocal microscopy. Neu2 was stained with a specific polyclonal antibody (Neu2 antibody PA5-35114) (red) at indicated time points (hpt). Untreated cells were used as a control for basal Neu2 expression. Nuclei stained with *Hoechst* (blue). Images (20X) are representative of three independent experiments. Scale bar, 10 μM.
(TIF)

S12 Fig Related to Fig 7E: NS1 protein from all four DENV serotypes but not WNV increases the expression of Neu3 in HPMEC. Neu3 expression in HPMEC monolayers after treatment with NS1 protein from DENV1-4 or WNV (5 μg/ml), examined by confocal microscopy. Neu3 was stained with a specific polyclonal antibody (Ganglioside sialidase antibody (N-18): sc-55826) (yellow) at indicated time points (hpt). Untreated cells were used as a control for basal Neu3 expression. Nuclei stained with *Hoechst* (blue). Images (20X) are representative of three independent experiments. Scale bar, 10 μM.
(TIF)

S13 Fig Related to Fig 7F: NS1 protein from all four DENV serotypes but not WNV increases the surface staining of syndecan-1 in the EGL of HPMEC. Staining of syndecan-1 (green) on the surface of HPMEC monolayers over time (hpt) after treatment with NS1 protein from DENV1-4 or WNV (5 μg/ml), examined by confocal microscopy. Untreated cells were used as a control for basal syndecan-1 expression. Nuclei are stained with *Hoechst* (blue). Images are representative of three individual experiments (20X). Scale bar, 10 μM.
(TIF)

S14 Fig Related to Fig 7G: NS1 from all four DENV serotypes triggers the activation/increased expression of endothelial heparanase in HPMEC. Heparanase expression (red) in HPMEC monolayers over time (hpt) after treatment with protein from DENV1-4 or WNV (5 μg/ml), examined by confocal microscopy. Untreated cells were used as a control for basal heparanase expression. Nuclei stained with *Hoechst* (blue). Images are representative of three individual experiments (20X). Scale bar, 10 μM.
(TIF)

S15 Fig Related to Fig 7H: NS1 from all four DENV serotypes increases the activity of cathepsin L protease in HPMEC. Cathepsin L proteolytic activity (Magic Red assay, in red) in HPMEC monolayers over time (hpt) after treatment with NS1 protein from DENV1-4 or WNV (5 μg/ml). Nuclei are stained with *Hoechst* (blue). Untreated cells were used as control for basal cathepsin L expression. Images are representative of three individual experiments (20X).
(TIF)
