# Supplementary figures and images for: Dengue Virus NS1 Disrupts the Endothelial Glycocalyx, Leading to Hyperpermeability
Source: PLoS Pathog. 2016 Jul 14;12(7):e1005738. doi: 10.1371/journal.ppat.1005738 (PMC4944995; doi:10.1371/journal.ppat.1005738)

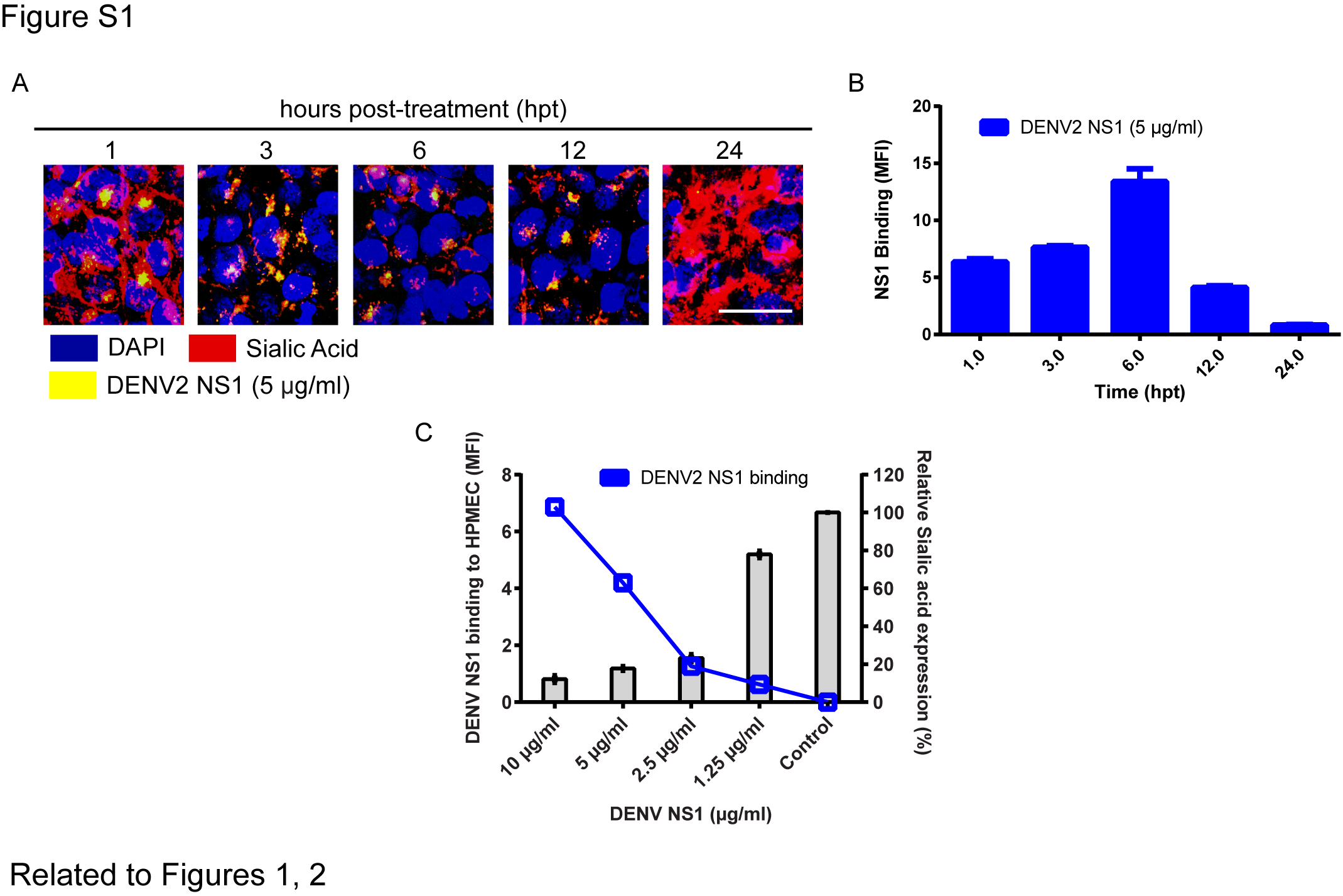

Supplement: S1 Fig — (A) Binding of DENV2 NS1 to HPMEC monolayers, examined by confocal microscopy. DENV2 NS1 was stained with a specific monoclonal antibody (9NS1 conjugated to Alexa 488), and Sia was stained with WGA-A647 (red) at indicated time points (hpt). Nuclei stained with Hoechst (blue). Images (20X) are representative of three independent experiments. Scale bar, 10 μM. (B) Quantification of MFI of DENV2 NS1 staining in S1B Fig from three independent experiments. (C) DENV2 NS1 disrupts Sia on the EGL of HPMEC in a dose-dependent manner. Quantification of Sia expression on cell surface and DENV2 NS1 binding to HPMEC monolayers in the presence of different concentrations of DENV2 NS1 (1.25, 2.5, 5, and 10 μg/ml) in Fig 2C. Results represent mean fluorescence intensity (MFI) values from three independent experiments. Grey bars represent relative sialic acid expression, calculated by normalizing each condition to untreated control cells (relative sialic acid expression = sialic acid expression in DENV2 NS1-treated monolayers/sialic acid expression in untreated control monolayers). The blue line represents DENV2 NS1 binding. (TIF) [file ppat.1005738.s001.tif]

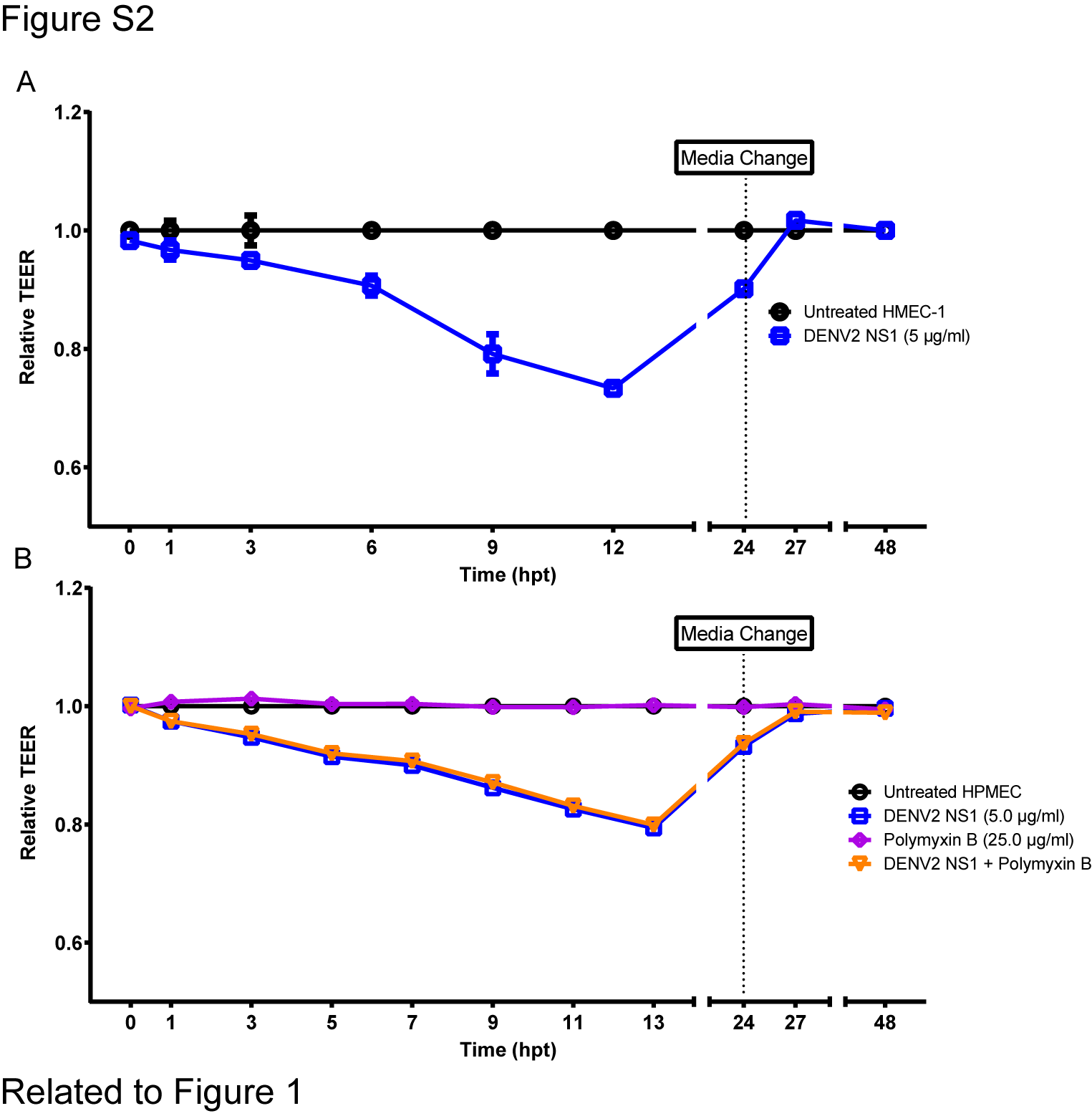

Supplement: S2 Fig — (A) TEER assay to evaluate the effect of DENV2 NS1 (5 μg/ml) on HMEC-1 endothelial permeability. Relative TEER values from one independent experiment performed in duplicate are plotted at the indicated time points. Error bars indicate standard error of the mean (SEM). DENV2 induces statistically significant decreases in TEER (p<0.05). (B) Effect of polymyxin B on DENV2 NS1-mediated endothelial hyperpermeability (TEER) in HPMEC monolayers. Relative TEER values from three independent experiments performed in duplicate are plotted at indicated time points (hpt). TEER values for monolayers treated with DENV2 NS1 combined with polymyxin B (25 μg/ml) are not significantly different from monolayers treated with DENV2 NS1 alone. Error bars indicate SEM throughout. (TIF) [file ppat.1005738.s002.tif]

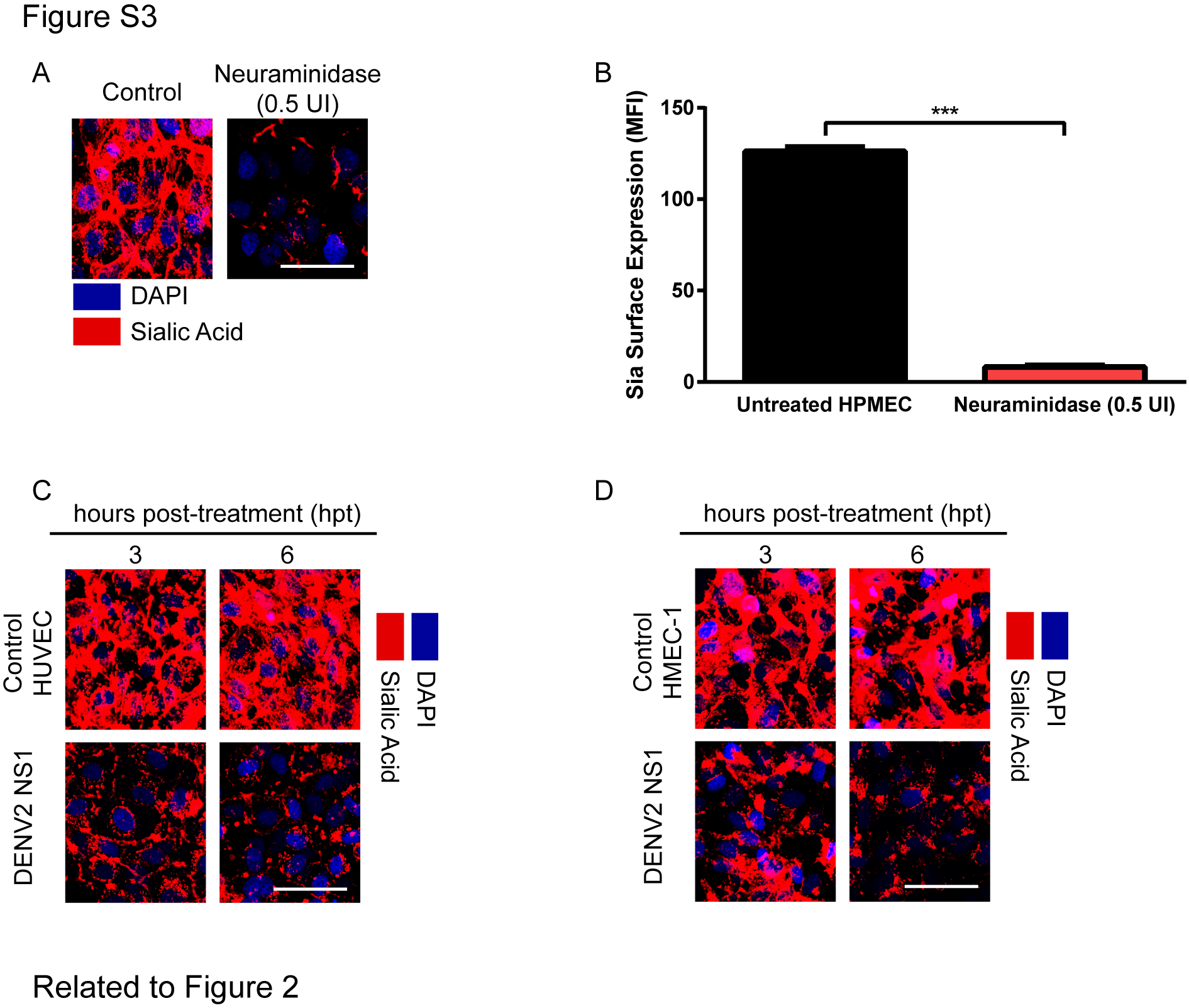

Supplement: S3 Fig — (A) Sia expression on HPMEC monolayers after treatment with recombinant neuraminidase from Clostridium perfringens (0.5 UI), examined by confocal microscopy. Sia was stained with WGA-A647 (red) at indicated time points (hpt). Untreated cells were used as a control for basal Sia expression. Nuclei stained with Hoechst (blue). Images (20X) are representative of three independent experiments. Scale bar, 10 μM. (B) Quantification of MFI in S3A Fig from three independent experiments. Sia expression in monolayers treated with recombinant neuraminidase is significantly different than in untreated control monolayers (p<0.0001). (C) Sia expression on HUVEC monolayers after treatment with DENV2 NS1 (5 μg/ml), examined by confocal microscopy. Sia was stained with WGA-A647 (red) at indicated time points (hpt). Nuclei are stained with Hoechst (blue). Untreated cells were used as control for basal Sia expression. Images are representative of one individual experiment (20X). (D) Sia expression on HMEC-1 monolayers after treatment with DENV2 NS1 (5 μg/ml), examined by confocal microscopy. Sia was stained with WGA-A647 (red) at indicated time points (hpt). Nuclei are stained with Hoechst (blue). Untreated cells were used as control for basal Sia expression. Images are representative of one individual experiment (20X). (TIF) [file ppat.1005738.s003.tif]

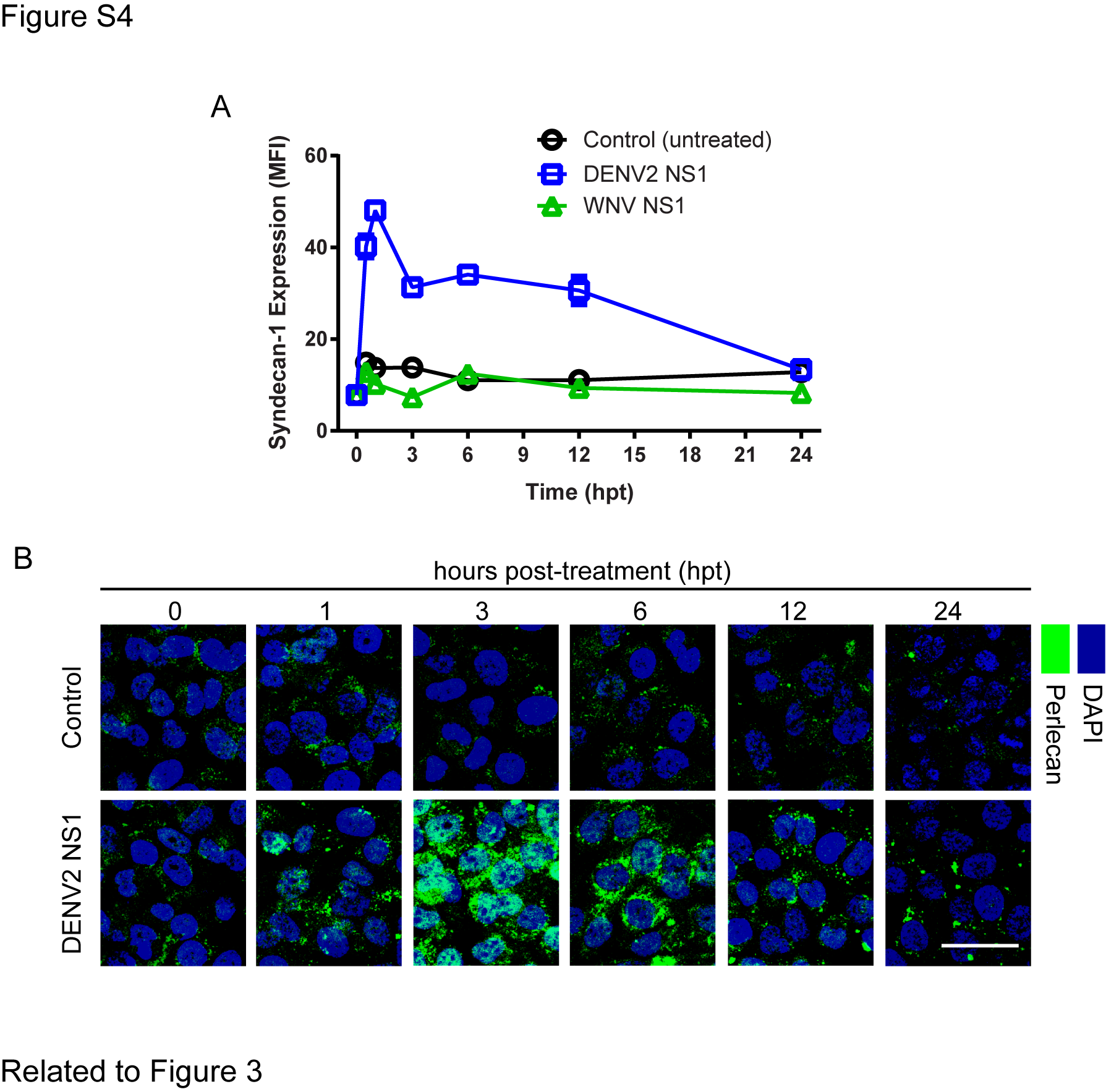

Supplement: S4 Fig — (A) Quantification of syndecan-1 MFI in Fig 3A. Staining is significantly higher with DENV2 NS1 compared to controls at 0.5–12 hpt (p<0.0001). Error bars indicate SEM. (B) Expression of perlecan (green) on the surface of HPMEC monolayers over time (hpt) after treatment with DENV2 or WNV NS1 proteins (5 μg/ml), examined by confocal microscopy. Untreated cells were used as a control for basal perlecan expression. Nuclei are stained with Hoechst (blue). Images are representative of three individual experiments (20X). Scale bar, 10 μM. A trace of the MFI of one representative field is shown below each image. (TIF) [file ppat.1005738.s004.tif]

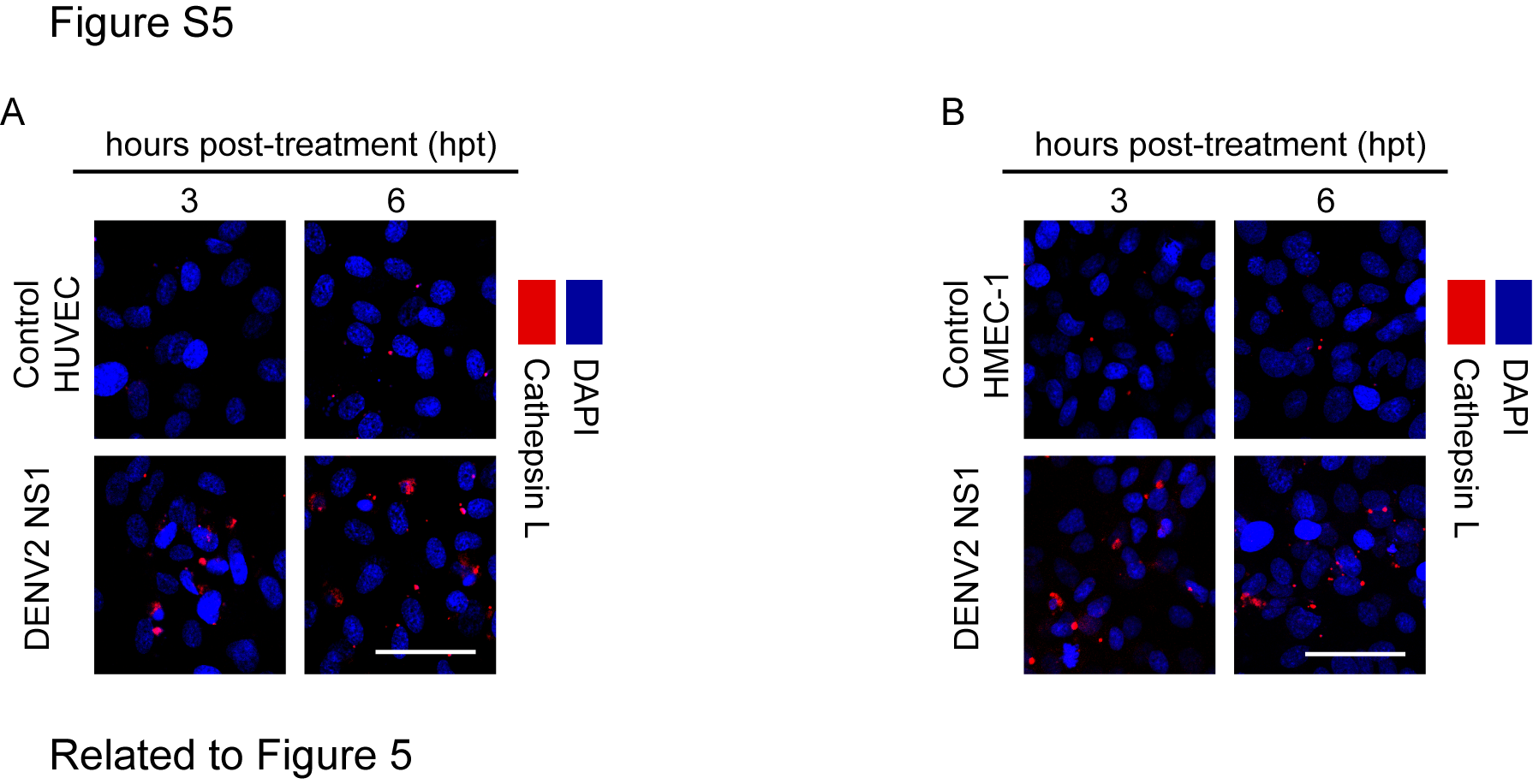

Supplement: S5 Fig — (A) Cathepsin L proteolytic activity (Magic Red assay, in red) in HUVEC monolayers over time (hpt) after treatment with DENV2 NS1 (5 μg/ml). Nuclei are stained with Hoechst (blue). Untreated cells were used as control for basal cathepsin L expression. Images are representative of one individual experiment (20X). (B) Cathepsin L proteolytic activity (Magic Red assay, in red) in HMEC-1 monolayers over time (hpt) after treatment with DENV2 NS1 (5 μg/ml). Nuclei are stained with Hoechst (blue). Untreated cells were used as control for basal cathepsin L expression. Images are representative of one experiment (20X). (TIF) [file ppat.1005738.s005.tif]

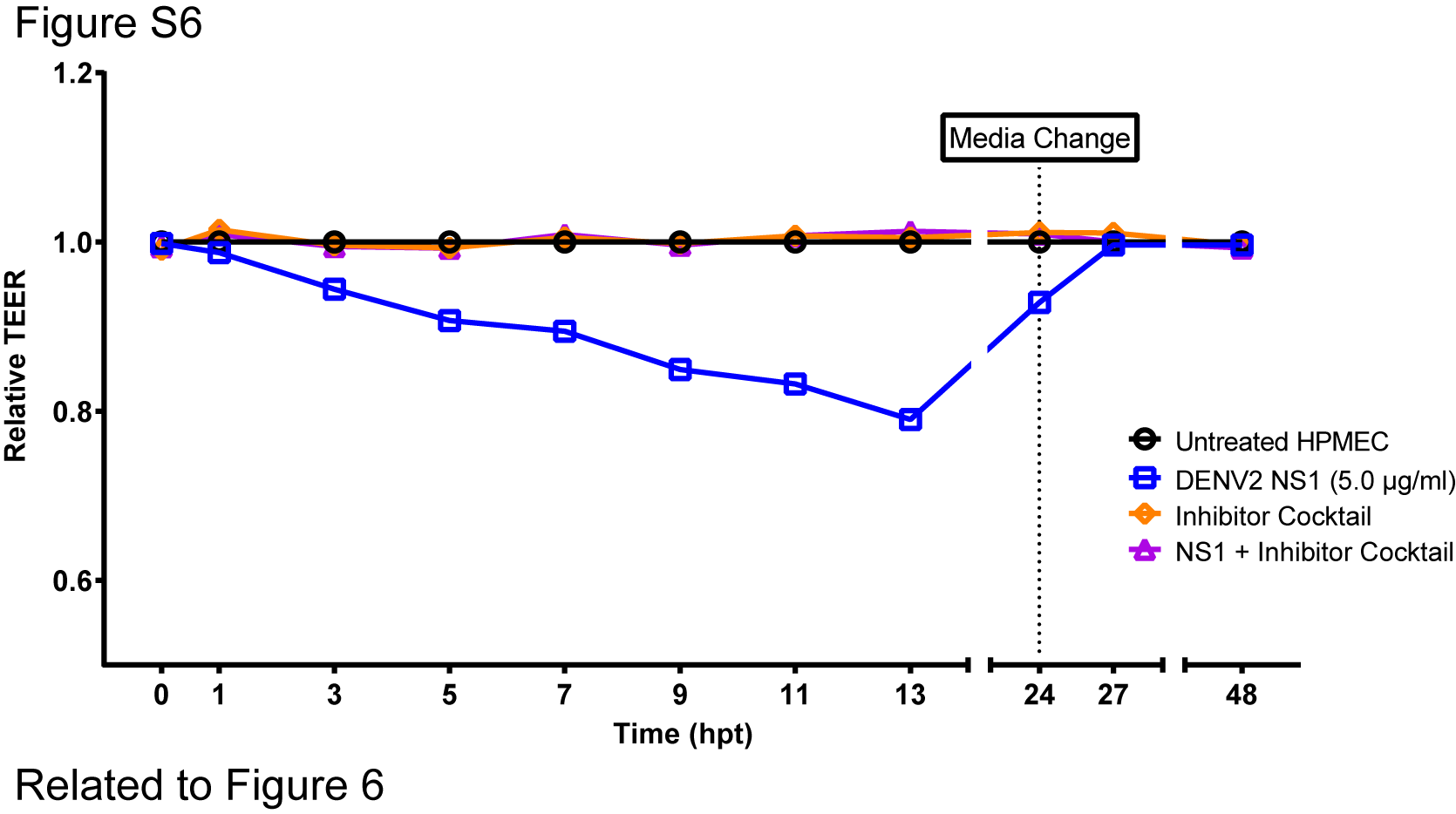

Supplement: S6 Fig — Effect of an inhibitor cocktail (DANA, 50 μg/ml; OGT 2115, 1.0 μM; cathepsin L inhibitor, 10 μM) on DENV2 NS1-triggered endothelial hyperpermeability (TEER) in HPMEC monolayers. TEER values of monolayers treated with DENV2 NS1 plus the inhibitor cocktail are significantly different than values of monolayers treated with DENV2 NS1 alone (p<0.0001) and not significant compared to untreated control monolayers. (TIF) [file ppat.1005738.s006.tif]

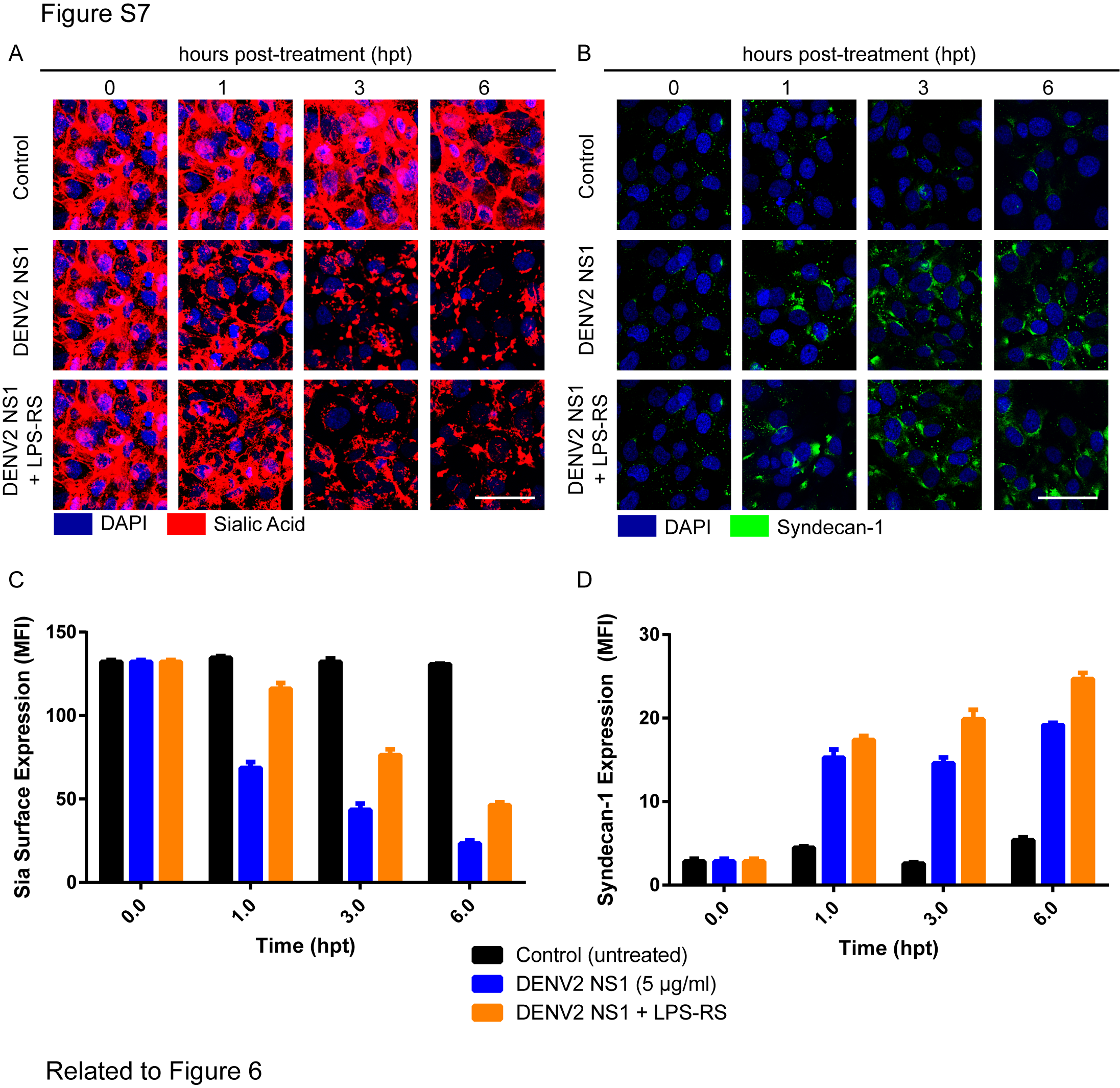

Supplement: S7 Fig — (A) Sia expression on HPMEC monolayers after treatment with DENV2 NS1 (5 μg/ml) and LPS-RS (50 μg/ml), examined by confocal microscopy. Sia was stained with WGA-A647 (red) at indicated time points (hpt). Untreated cells were used as a control for basal Sia expression. Nuclei stained with Hoechst (blue). Images (20X) are representative of three independent experiments. Scale bar, 10 μM. (B) Staining of syndecan-1 (green) on the surface of HPMEC monolayers over time (hpt) after treatment with DENV2 NS1 (5 μg/ml) and LPS-RS (50 μg/ml), examined by confocal microscopy. Untreated cells were used as a control for basal syndecan-1 expression. Nuclei are stained with Hoechst (blue). Images are representative of three individual experiments (20X). Scale bar, 10 μM. (C) Quantification of MFI in S12A Fig from three independent experiments. Sia expression in monolayers treated with DENV2 NS1 and LPS-RS is significantly higher than in monolayers treated with only DENV2 NS1 at 1, 3, and 6 hpt (p<0.0001). (D) Quantification of MFI in S12B Fig from three independent experiments. Syndecan-1 expression in monolayers treated with DENV2 NS1 and LPS-RS is significantly higher than in monolayers treated with only DENV2 NS1 at 3 and 6 hpt (p<0.0001). (TIF) [file ppat.1005738.s007.tif]

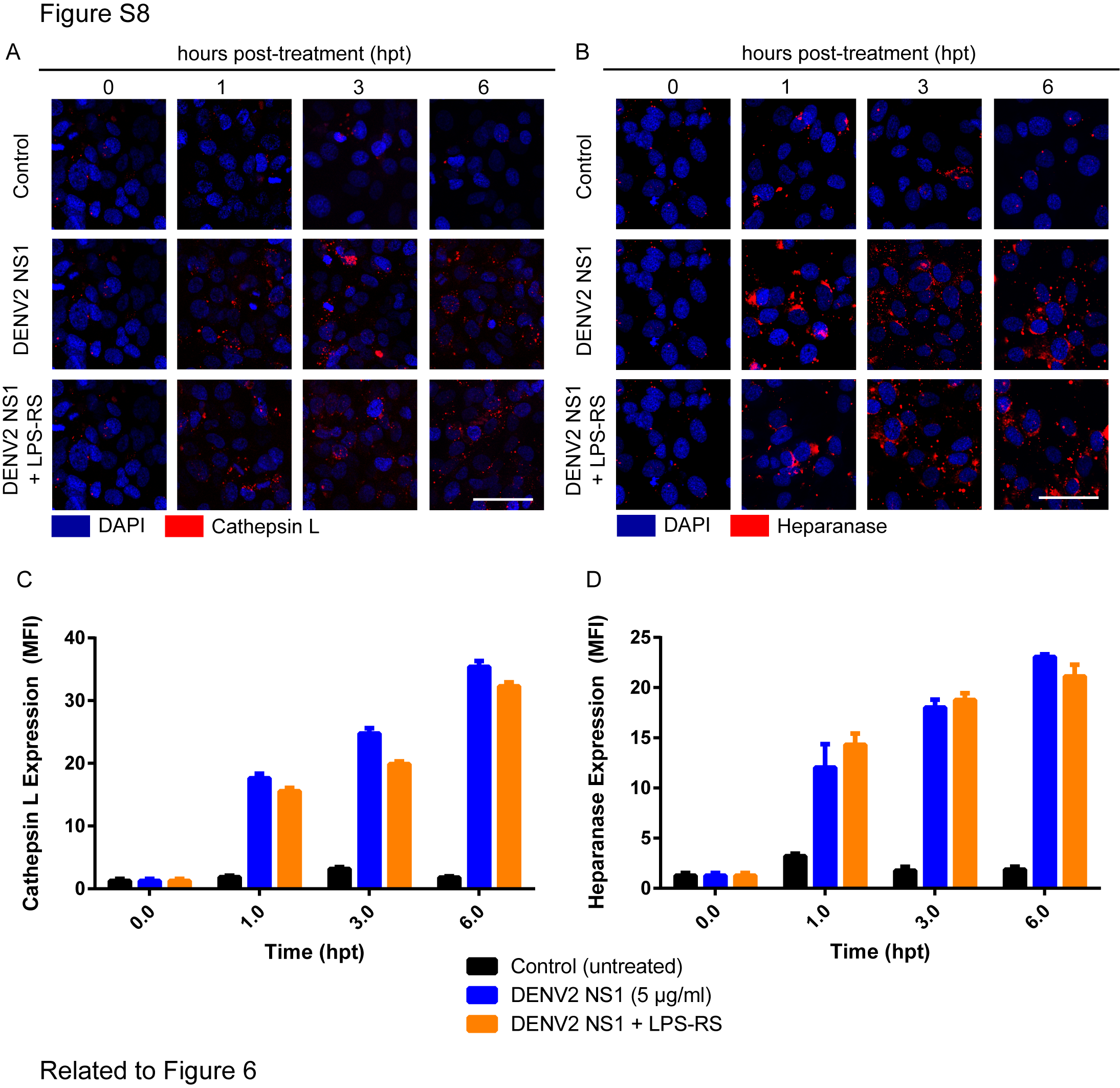

Supplement: S8 Fig — (A) Cathepsin L proteolytic activity (Magic Red assay, in red) in HPMEC monolayers over time (hpt) after treatment with DENV2 NS1 (5 μg/ml) and LPS-RS, examined by confocal microscopy. Nuclei are stained with Hoechst (blue). Untreated cells were used as control for basal cathepsin L expression. Images are representative of three individual experiments (20X). (B) Heparanase expression (red) in HPMEC monolayers over time (hpt) after treatment with DENV2 NS1 (5 μg/ml) and LPS-RS, examined by confocal microscopy. Untreated cells were used as a control for basal heparanase expression. Nuclei stained with Hoechst (blue). Images are representative of three individual experiments (20X). Scale bar, 10 μM. (C) Quantification of MFI in S13B Fig from three independent experiments. Cathepsin L activity in monolayers treated with DENV2 NS1 and LPS-RS is significantly lower than in monolayers treated with only DENV2 NS1 at 3 and 6 hpt (3 hpt, p<0.0001; 6pht, p<0.005). (D) Quantification of MFI in S13A Fig from three independent experiments. Heparanase expression in monolayers treated with DENV2 NS1 and LPS-RS is not significantly different than in monolayers treated with only DENV2 NS1. (TIF) [file ppat.1005738.s008.tif]

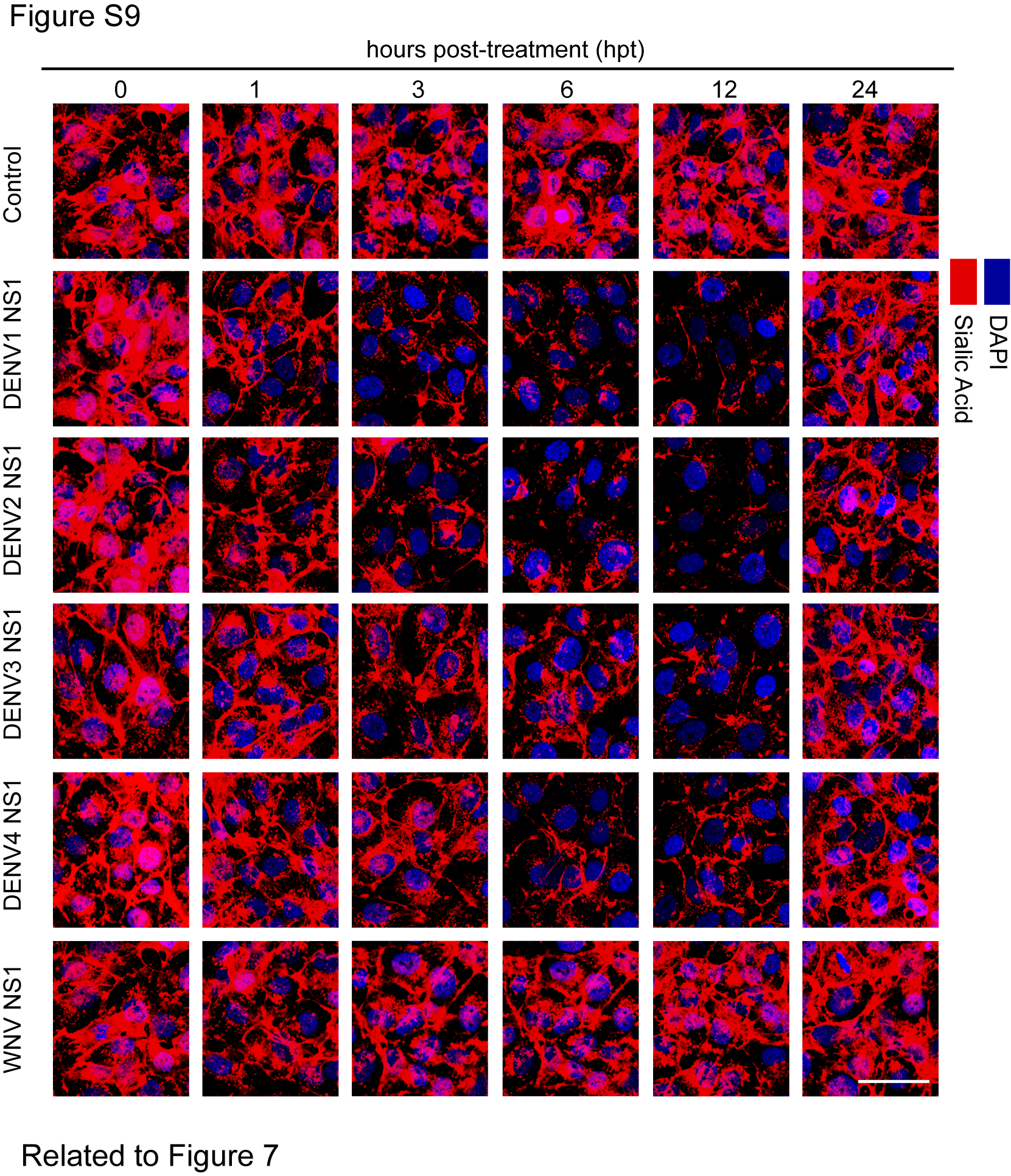

Supplement: S9 Fig — Sia expression on HPMEC monolayers after treatment with NS1 protein from DENV1-4 or WNV (5 μg/ml), examined by confocal microscopy. Sia was stained with WGA-A647 (red) at indicated time points (hpt). Untreated cells were used as a control for basal Sia expression. Nuclei stained with Hoechst (blue). Images (20X) are representative of three independent experiments. Scale bar, 10 μM. (TIF) [file ppat.1005738.s009.tif]

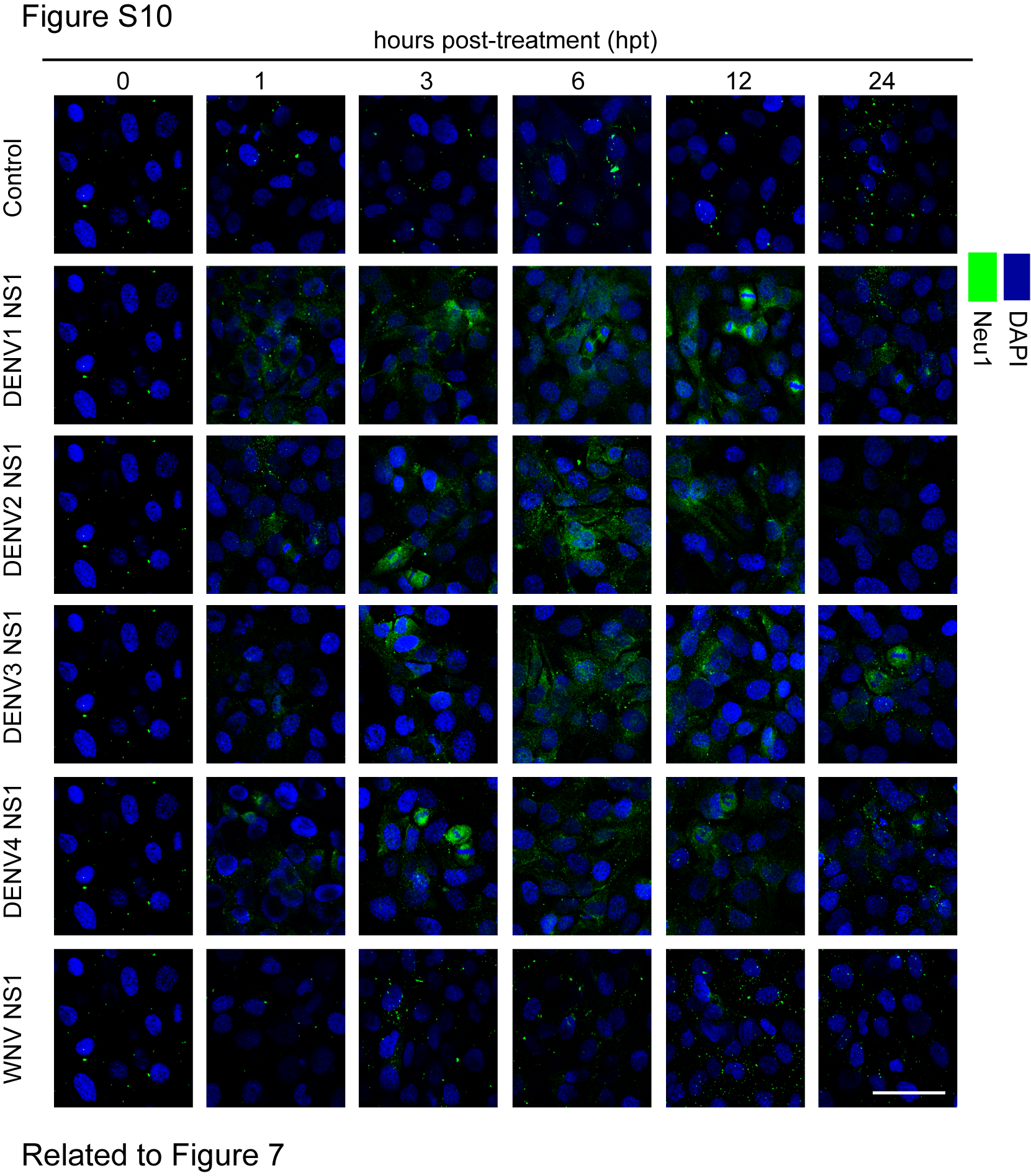

Supplement: S10 Fig — Neu1 expression in HPMEC monolayers after treatment with NS1 protein from DENV1-4 or WNV (5 μg/ml), examined by confocal microscopy. Neu1 was stained with a specific polyclonal antibody (Neu1 antibody (H-300): sc-32936) (green) at indicated time points (hpt). Untreated cells were used as a control for basal Neu1 expression. Nuclei stained with Hoechst (blue). Images (20X) are representative of three independent experiments. Scale bar, 10 μM. (TIF) [file ppat.1005738.s010.tif]

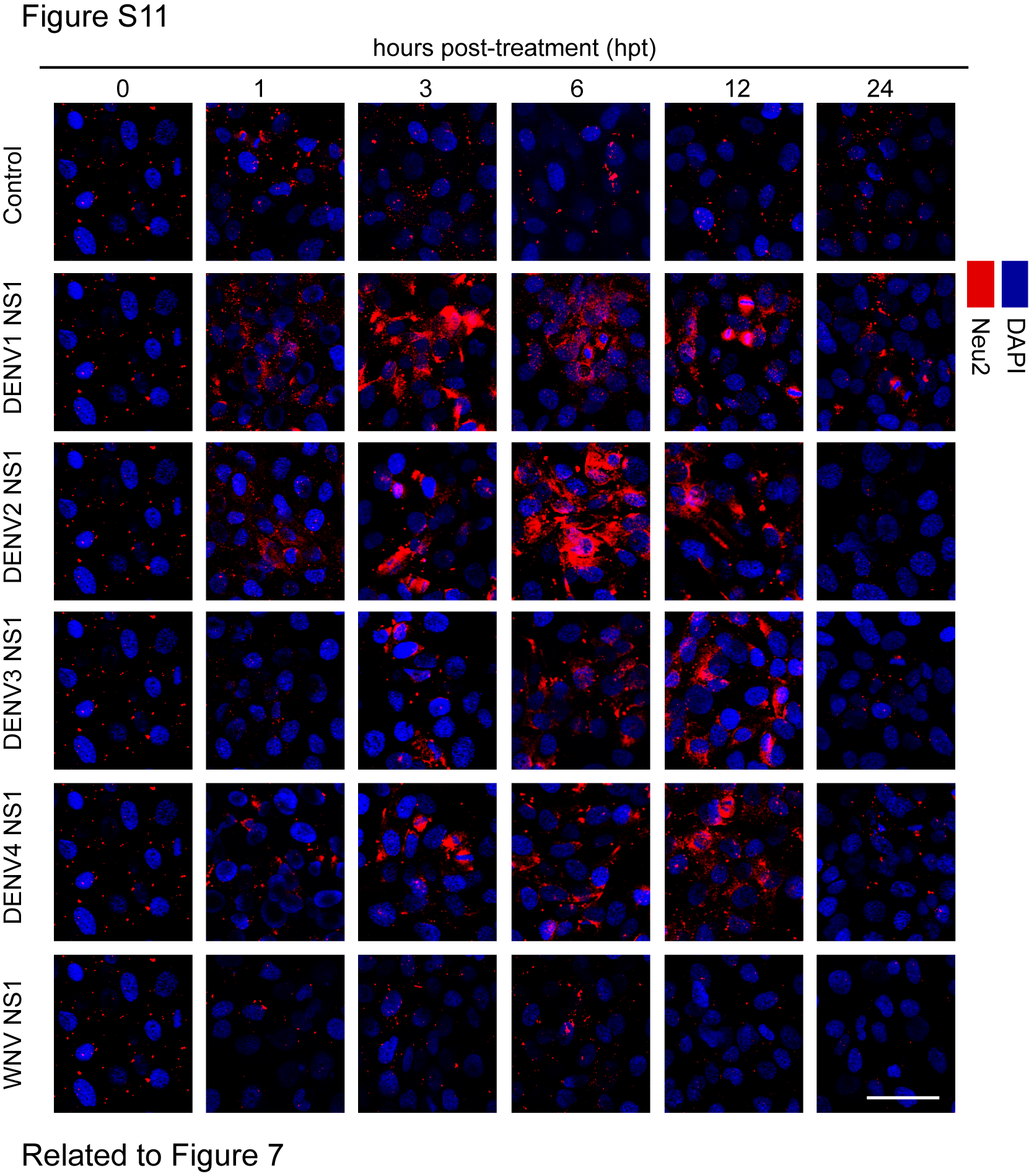

Supplement: S11 Fig — Neu2 expression in HPMEC monolayers after treatment with NS1 protein from DENV1-4 or WNV (5 μg/ml), examined by confocal microscopy. Neu2 was stained with a specific polyclonal antibody (Neu2 antibody PA5-35114) (red) at indicated time points (hpt). Untreated cells were used as a control for basal Neu2 expression. Nuclei stained with Hoechst (blue). Images (20X) are representative of three independent experiments. Scale bar, 10 μM. (TIF) [file ppat.1005738.s011.tif]

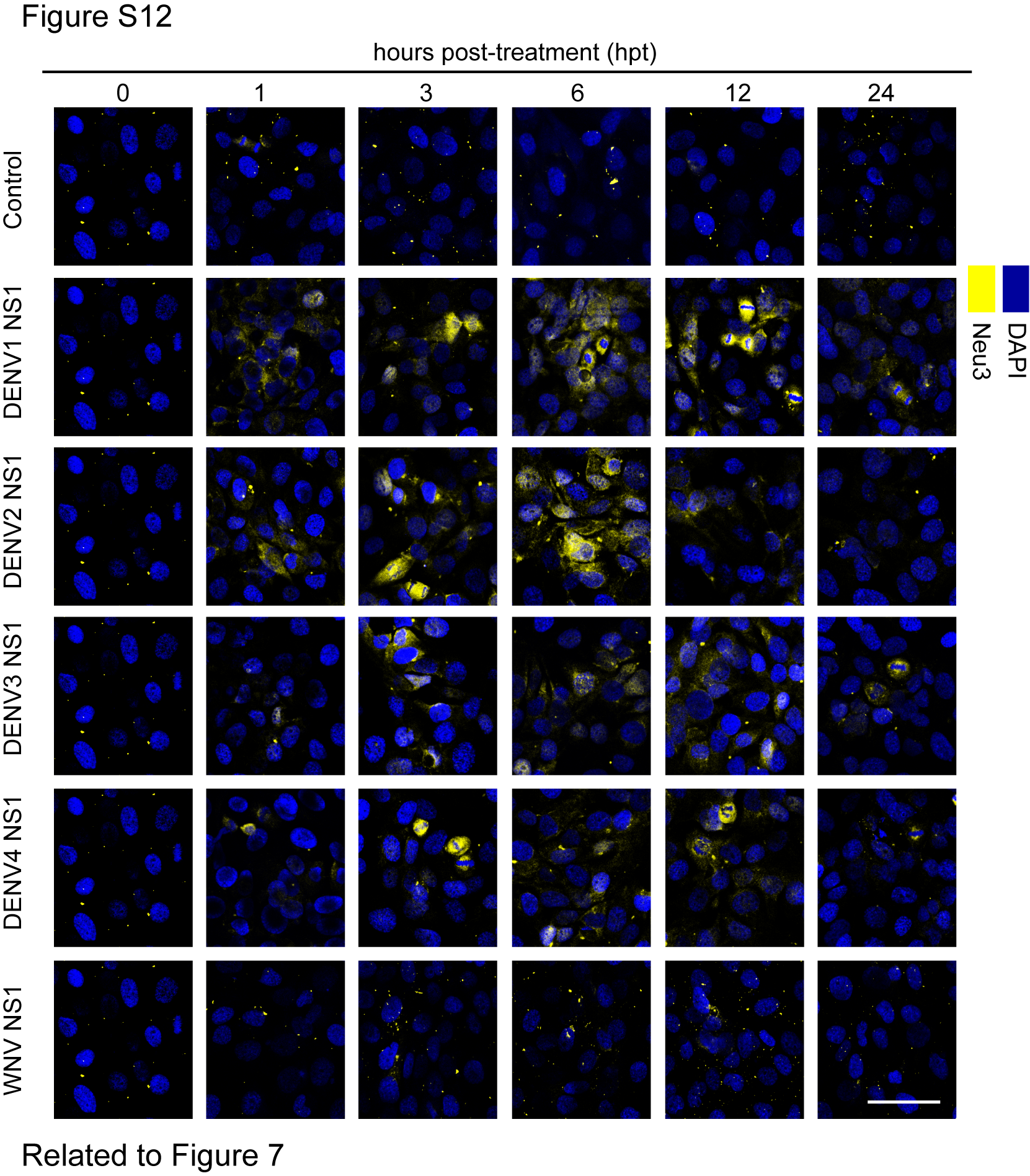

Supplement: S12 Fig — Neu3 expression in HPMEC monolayers after treatment with NS1 protein from DENV1-4 or WNV (5 μg/ml), examined by confocal microscopy. Neu3 was stained with a specific polyclonal antibody (Ganglioside sialidase antibody (N-18): sc-55826) (yellow) at indicated time points (hpt). Untreated cells were used as a control for basal Neu3 expression. Nuclei stained with Hoechst (blue). Images (20X) are representative of three independent experiments. Scale bar, 10 μM. (TIF) [file ppat.1005738.s012.tif]

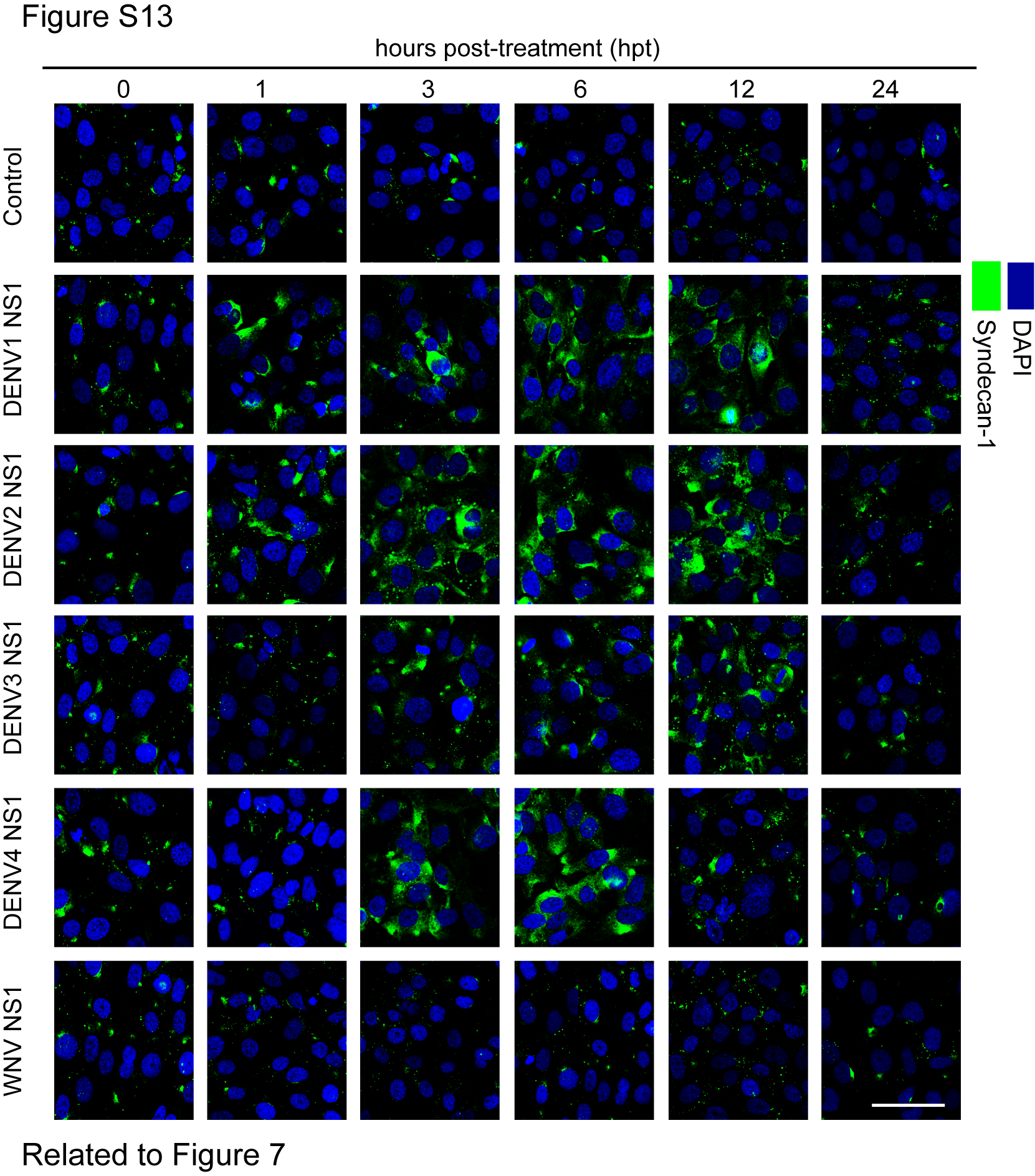

Supplement: S13 Fig — Staining of syndecan-1 (green) on the surface of HPMEC monolayers over time (hpt) after treatment with NS1 protein from DENV1-4 or WNV (5 μg/ml), examined by confocal microscopy. Untreated cells were used as a control for basal syndecan-1 expression. Nuclei are stained with Hoechst (blue). Images are representative of three individual experiments (20X). Scale bar, 10 μM. (TIF) [file ppat.1005738.s013.tif]

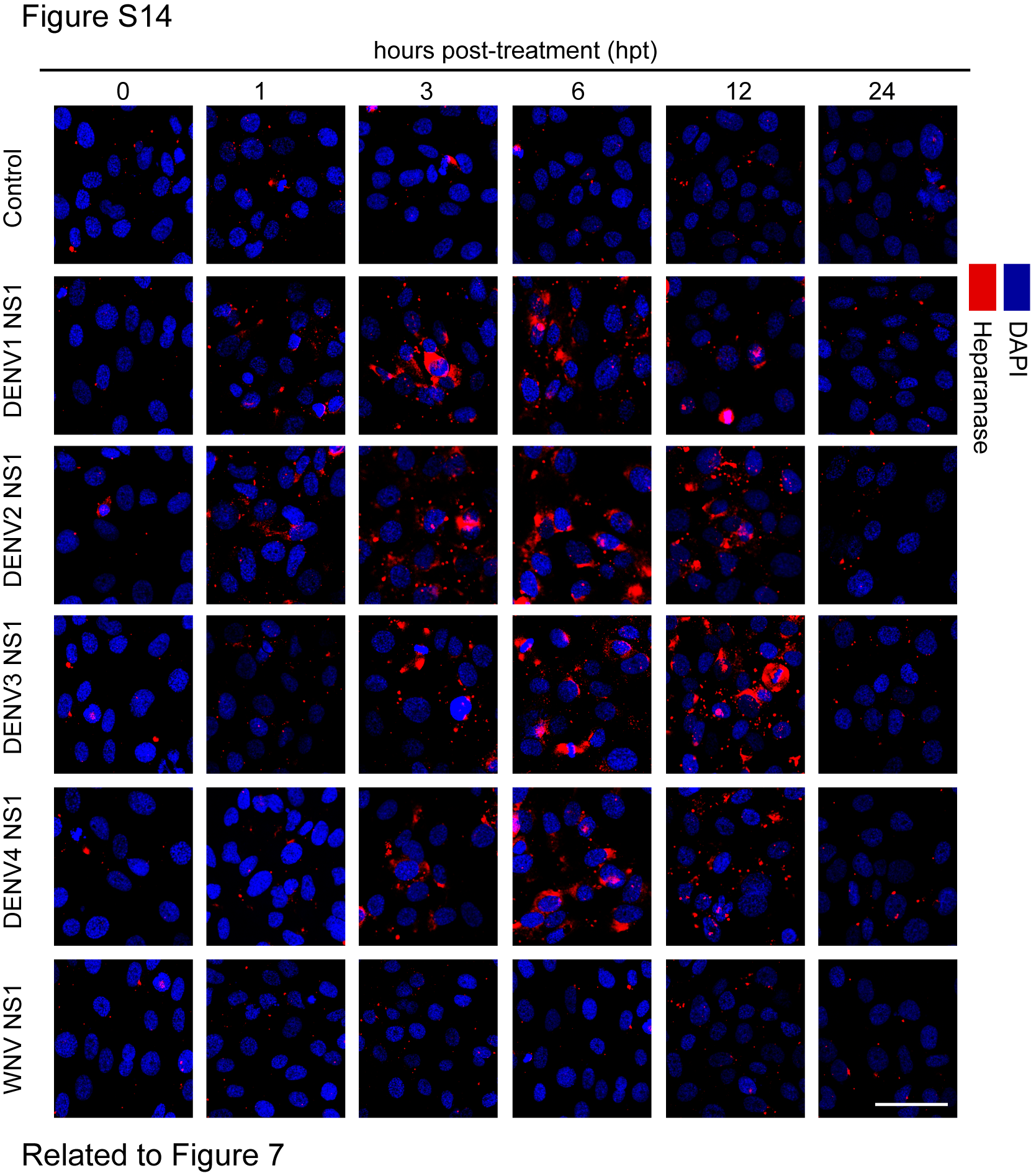

Supplement: S14 Fig — Heparanase expression (red) in HPMEC monolayers over time (hpt) after treatment with protein from DENV1-4 or WNV (5 μg/ml), examined by confocal microscopy. Untreated cells were used as a control for basal heparanase expression. Nuclei stained with Hoechst (blue). Images are representative of three individual experiments (20X). Scale bar, 10 μM. (TIF) [file ppat.1005738.s014.tif]

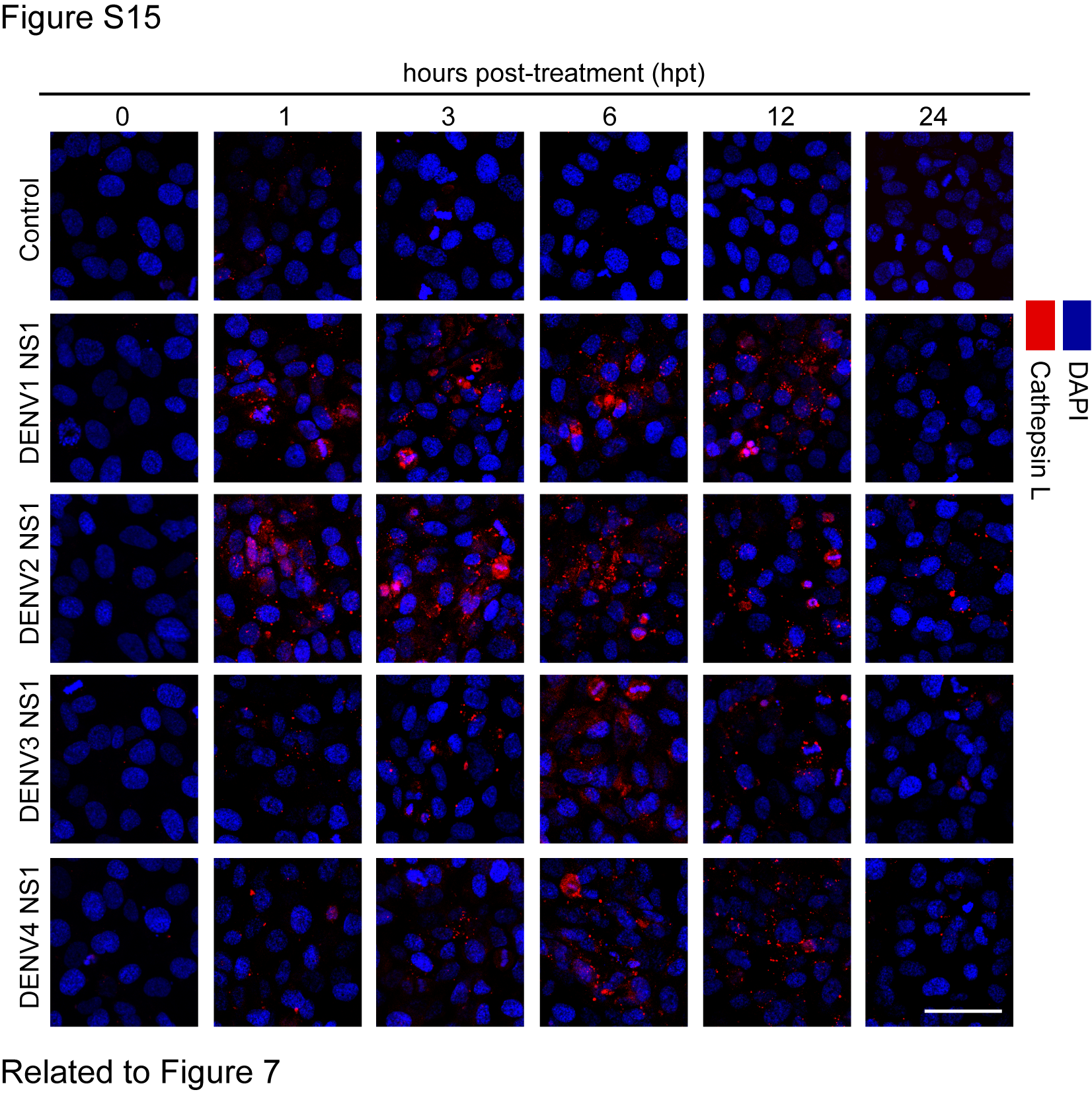

Supplement: S15 Fig — Cathepsin L proteolytic activity (Magic Red assay, in red) in HPMEC monolayers over time (hpt) after treatment with NS1 protein from DENV1-4 or WNV (5 μg/ml). Nuclei are stained with Hoechst (blue). Untreated cells were used as control for basal cathepsin L expression. Images are representative of three individual experiments (20X). (TIF) [file ppat.1005738.s015.tif]
